# An Efficient Approach for the Design and Synthesis of Antimicrobial Peptide-Peptide Nucleic Acid Conjugates

**DOI:** 10.3389/fchem.2022.843163

**Published:** 2022-03-15

**Authors:** Nitin A. Patil, Varsha J. Thombare, Rong Li, Xiaoji He, Jing Lu, Heidi H. Yu, Hasini Wickremasinghe, Kavya Pamulapati, Mohammad A. K. Azad, Tony Velkov, Kade D. Roberts, Jian Li

**Affiliations:** ^1^ Infection and Immunity Program and Department of Microbiology, Biomedicine Discovery Institute, Monash University, Melbourne, VIC, Australia; ^2^ Department of Biochemistry and Pharmacology, The University of Melbourne, Melbourne, VIC, Australia

**Keywords:** antisense oligonucleotides, peptide nucleic acids, antimicrobial agents, cell-penetrating peptides, conjugation

## Abstract

Peptide-Peptide Nucleic Acid (PNA) conjugates targeting essential bacterial genes have shown significant potential in developing novel antisense antimicrobials. The majority of efforts in this area are focused on identifying different PNA targets and the selection of peptides to deliver the peptide-PNA conjugates to Gram-negative bacteria. Notably, the selection of a linkage strategy to form peptide-PNA conjugate plays an important role in the effective delivery of PNAs. Recently, a unique Cysteine- 2-Cyanoisonicotinamide (Cys-CINA) click chemistry has been employed for the synthesis of cyclic peptides. Considering the high selectivity of this chemistry, we investigated the efficiency of Cys-CINA conjugation to synthesize novel antimicrobial peptide-PNA conjugates. The PNA targeting acyl carrier protein gene (*acpP*), when conjugated to the membrane-active antimicrobial peptides (polymyxin), showed improvement in antimicrobial activity against multidrug-resistant Gram-negative *Acinetobacter baumannii*. Thus, indicating that the Cys-CINA conjugation is an effective strategy to link the antisense oligonucleotides with antimicrobial peptides. Therefore, the Cys-CINA conjugation opens an exciting prospect for antimicrobial drug development.

## Introduction

**FIGURE 1:**
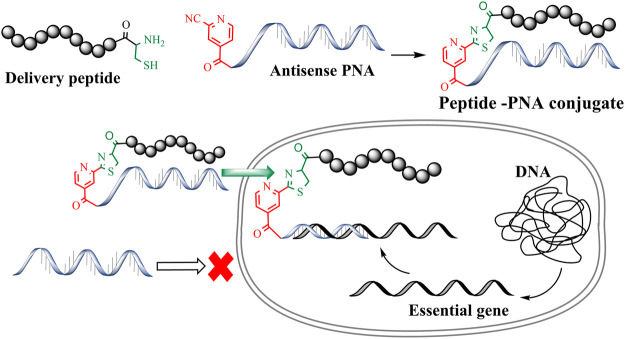
CINA mediated conjugation to generate peptide-PNA antisense conjugates.

Antimicrobial resistance is now considered one of the greatest threats to global health and the economy. The World Health Organization (WHO) identified multidrug-resistant (MDR) Gram-negative pathogens such as *Pseudomonas aeruginosa, Acinetobacter baumannii, Enterobacterales* as the top priority for the development of new antibiotics (WHO, 2017). This situation has been made even more problematic by the lack of development of new effective antibiotic drug therapies targeting these multi-drug resistant (MDR) bacteria. Therefore, there is an urgent requirement for the development of antibiotics with novel modes of action (Andersson et al., 2016; WHO, 2017). To this end, antisense antibiotics such as Peptide Nucleic Acids (PNAs) can be used to target essential genes in bacteria, resulting in translational gene silencing and bactericidal effects (Good et al., 2001; Ghosal and Nielsen, 2012). PNAs are artificial DNA mimics with tertiary amide linked nucleobases and aminoethylglycine (*aeg*) repeating units (Nielsen et al., 1991; Egholm et al., 1993; Wittung et al., 1996; Good and Nielsen, 1998; Nielsen, 2001). This unique combination of amide linkage and carbonyl methylene linked nucleobases provides thermal and hydrolytic (enzymatic degradation) stability. Furthermore, PNAs are easy to synthesize and exhibit improved binding affinity towards complementary DNA/RNA sequences. The last decade has seen a rapid surge in the development of PNA-based therapeutics and biochemical tools (Siddiquee et al., 2015; Gupta et al., 2017). However, the charge-neutral amide backbone in PNA can result in poor cell permeability that leads to poor antibacterial activity (Koppelhus and Nielsen, 2003). Conjugation of PNAs with cell-penetrating peptides (CPP) is a widely used approach to improve the cellular permeability of antisense PNAs (Mcallister et al., 2006; Gupta et al., 2016). Importantly, CPP mediated intracellular delivery of oligonucleotides has been effectively used to target essential bacterial genes and develop novel antimicrobial peptide-PNA conjugates (Figure 1) (Good et al., 2001; Ghosal et al., 2013). Along these lines, peptide-mediated cell penetration was shown to be effective for the delivery of antisense PNA targeting top-priority *Acinetobacter baumannii* (Rose et al., 2019).

A number of conjugation strategies have been explored to link PNA with CPPs, including disulfide (Turner et al., 2006), thio-maleimide (Ede et al., 1994) and triazole (McKay and Finn, 2014). However, these strategies are often limited to simple and linear CPPs. Importantly, conjugation of PNAs with structurally complex peptides such as the polymyxins (Velkov et al., 2013) and cathelicidin (Dürr et al., 2006) remains an unexplored terrain (Soudah et al., 2019). Moreover, peptide conjugations through traditional disulfide and maleimide linkages require orthogonal protecting groups as well as a multistep synthesis strategy (Patil, 2021). Previously, we developed a methodology for cysteine-cyanobenzothiazole (Cys-CBT) mediated peptide-PNA conjugation that enables facile synthesis and improved intracellular stability as compared to other strategies (Patil et al., 2019). However, commercially available 2-cyanobenzothiazole is expensive and requires additional synthetic steps before being employed for the solid-phase synthesis of peptides or PNAs. In our hands, succinoyl-2-cyanobenzothiazole moiety increased the hydrophobicity of peptide and PNA analogues, resulting in conjugates with poor aqueous solubility (Patil et al., 2019; Patil et al., 2021). To overcome the high-cost and hydrophobicity limitations of the previously developed Cys-CBT conjugation method, we have exploited the low-cost commercially avaliable 2-cyanoisonicotinamide (CINA) group (Patil et al., 2021). This conjugation strategy involves reaction between an *N*-terminal cysteine residue and the nitrile group of cyanoisonicotinic acid which forms a thiazole ring (Figure 1). The resulting linkage is less hydrophobic due to the presence of smaller heterocyclic moiety. As an improved strategy, Cys-CINA conjugation utilizes a relatively water-soluble, readily available CINA moiety and retains all the other biological advantages of Cys-CBT linkage. We have previously utilized this chemo-selective chemistry for peptide macrocyclization (Patil et al., 2021). In the present work we explore the utility of the Cys-CINA conjugation strategy for the generation of novel antimicrobial peptide-PNA conjugates.

## Materials and Methods

### Materials

Piperidine, Trifluoroacetic acid (TFA) and 1H-Benzotriazolium-1-[bis(dimethylamino)methylene]-5-chloro-hexafluorophosphate-(1-),3-oxide (HCTU) were obtained from Auspep (Melbourne, Australia), and Fmoc**-**8-amino-3,6-dioxaoctanoic acid (PEG), Fmoc-amino acids were obtained from Chem-Impex International (United States). PNA monomers Fmoc-A (Bhoc)-OH, Fmoc-C(Bhoc)-OH, Fmoc-T (Bhoc)-OH, Fmoc-G (Bhoc)-OH were obtained from PANGENE Inc. (Daejeon, Republic of Korea)). Dimethylformamide (DMF), methanol (MeOH), diethyl ether, dichloromethane (DCM), hydrochloric acid (HCl) and acetonitrile were obtained from Merck (Melbourne, Australia). LC-MS grade water and acetonitrile were obtained from Fisher Chemicals (Melbourne, Australia). 2-Chlorotrityl-Resin and Rink amide resin were obtained from Chempep Inc. Wellington, United States. Triisopropylsilane (TIPS), 2,2′-(Ethylenedioxy)diethanethiol (DODT), diphenylphosphorylazide (DPPA), 2,2′-dithiodipyridine (DPDS), N,N′-Diisopropylcarbodiimide (DIC), and diisopropylethylamine (DIPEA), N-Methylpyrrolidone (NMP), propidium iodide and phenazine methosulfate were obtained from Sigma-Aldrich (Castle Hill, Australia). Dulbecco’s modified eagle medium (DMEM) was purchased from Gibco (Thermo Fisher Scientific, Waltham, MA, United States). Fetal bovine serum (FBS) was from Bovogen Biologicals (Victoria, Australia) and XTT staining reagent was obtained from Santa Cruz Biotechnology (Dallas, TX, United States).

### PNA Synthesis

All PNAs (Table 1) were synthesized using standard Fmoc chemistry. The PNA was synthesized on Rink amide resin (100–200, 0.25 mmol/g) 100 μmol scale. The Fmoc deprotection was conducted twice using piperidine 20% in DMF at room temperature for 3 min. The coupling of PNA monomers was performed using 3 molar equivalents of Fmoc-protected PNA(Bhoc)-OH monomers and HCTU dissolved in NMP, *in situ* activation using 6 molar equivalents of DIPEA for 30 min at room temperature. Following final PNA monomer coupling and deprotection, an 8-amino-3,6-dioxaoctanoic acyl (PEG) spacer and cysteine residue was coupled at the *N*-terminus (5′-end). All synthesized PNAs were cleaved from the solid support with a solution of TFA: H2O: TIPS (95:2.5:2.5, v/v/v). The *N*-terminal cysteine containing PNA (abbreviated below as Cys-PNA) was cleaved with an optimized solution of TFA: H2O: TIPS (95:2.5:2.5, v/v/v; 20 equivalent cysteamine) for 1.5 h. The TFA solution was filtered and evaporated under a nitrogen stream, and the PNA was precipitated in ice-cold diethyl ether; the PNA pellet was then washed twice with diethyl ether (40 ml). The crude PNA was analyzed with LC-MS (Method C) and purified by RP-HPLC method A. The fractions from RP-HPLC were combined and lyophilized for 2 days to give the purified peptides as their corresponding TFA salt. The purity of the PNA was confirmed by LC-MS analysis method D.

**TABLE 1:** Peptide-PNA conjugates sequences.

Conjugate ^c^	Peptide ^c^ and AA Sequence	PNA^c^ and nucleobase Sequence	Conjugation Yields
LASP-058	LASP-054	LASP-050	95%
	H-Arg-Acp-Arg-Arg-Acp-Arg-Arg-Acp-Arg-Arg-Acp-Arg-PEG-Lys (CINA)-NH_2_	Cys-PEG-Cyt-Thy-Cyt-Ade-Thy-Ade-Cyt-Thy-Cyt-Thy-Thy-Gua-Lys-NH_2_	
LASP-059	LASP-026	LASP-050	96%
	CINA-PEG-OctGly-Dab-Thr-Dab-Dab^b^-Dab-D-Phe-Leu-Dab-Dab-Thr^b^	Cys-PEG-Cyt-Thy-Cyt-Ade-Thy-Ade-Cyt-Thy-Cyt-Thy-Thy-Gua-Lys-NH_2_	
LASP-084	LASP-077	LASP-050	90%
	CINA-PEG-Gly-Lys-Pro-Arg-Pro-Tyr-Ser-Pro-Arg-Pro-Thr-Ser-His-Pro-Arg-Pro-Ile-Arg-Arg-NH_2_	Cys-PEG-Cyt-Thy-Cyt-Ade-Thy-Ade-Cyt-Thy-Cyt-Thy-Thy-Gua-Lys-NH_2_	
LASP-086	LASP-078	LASP-050	87%^a^
	CINA-PEG-Val-Cys^c^-Lys-Arg-trp-Lys-Lys-Trp-Lys-Arg-Lys-Trp-Lys-Lys-Trp-Cys^c^-Val-NH_2_	Cys-PEG-Cyt-Thy-Cyt-Ade-Thy-Ade-Cyt-Thy-Cyt-Thy-Thy-Gua-Lys-NH_2_	
LASP-088	LASP-078	LASP-050	92%
	CINA-PEG-Val-Cys^d^-Lys-Arg-trp-Lys-Lys-Trp-Lys-Arg-Lys-Trp-Lys-Lys-Trp-Cys^d^-Val-NH_2_	Cys-PEG-Cyt-Thy-Cyt-Ade-Thy-Ade-Cyt-Thy-Cyt-Thy-Thy-Gua-Lys-NH_2_	
LASP-130	LASP-072	LASP-095	94%
	Cys-PEG-OctGly-Dab-Thr-Dab-Dab^b^-Dab-D-Phe-Leu-Dab-Dab-Thr^b^	CINA-PEG-Cyt-Gua-Ade-Thy-Cyt-Ade-Thy-Thy-Cyt-Ade-Ade-Ade-Lys-NH_2_	
LASP-131	LASP-072	LASP-096	98%
	Cys-PEG-OctGly-Dab-Thr-Dab-Dab^b^-Dab-D-Phe-Leu-Dab-Dab-Thr^b^	CINA-PEG-Thy-Cyt-Cyt-Ade-Thy-Thy-Ade-Thy-Thy-Gau-Lys-NH_2_	
LASP-132	LASP-072	LASP-072	98%
	Cys-PEG-OctGly-Dab-Thr-Dab-Dab^b^-Dab-D-Phe-Leu-Dab-Dab-Thr^b^	Cys-PEG-OctGly-Dab-Thr-Dab-Dab^b^-Dab-D-Phe-Leu-Dab-Dab-Thr^b^	
LASP-133	LASP-097	LASP-119	96%
	CINA-PEG-Cyt-Thy-Cyt-Ade-Thy-Ade-Cyt-Thy-Cyt-Thy-Lys-NH_2_	CINA-PEG-Thy-Thy-Thy-Cyt-Thy-Cyt-Gua-Thy-Cyt-Ade-Lys-NH_2_	

a Conjugated and cross-linked yield.

b Dab side-chain and *C*-terminal cyclization.

c Side-chain cross-linked with a butylene bridge.

d Linked with a disulfide bridge.

### General Peptide Synthesis

All the peptides were synthesized using either Rink amide resin (100-200 mesh 0.61 mmol/g) or 2-Chlorotrityl Resin (0.1 mmol) (100–200 mesh, 0.71 mmol/g). The first amino acid on 2-Chlorotrityl Resin was loaded using 6 molar equivalents solution of respective Fmoc-amino acid in DMF. The rest of the amino acid sequence was built on a Prelude automated peptide synthesizer (Protein Technologies) using standard Fmoc solid-phase peptide chemistry. All coupling of the Fmoc-amino acids were performed using the default instrument protocol: 3 molar equivalents (relative to resin loading) of the Fmoc amino acid, 3 molar equivalents HCTU and 6 molar equivalents of DIPEA in DMF for 50 min at room temperature. Fmoc deprotection was performed using the default instrument protocol: 20% piperidine in DMF (1 × 5 min, 1 × 10 min) at room temperature. The crude peptide was taken up in 15 ml of TFA cleavage cocktail solution (2.5% DODT, 5% TIPS, 92.5% TFA) and stirred at room temperature for 90 min. TFA solution was filtered and evaporated under a nitrogen stream, then 40 ml of diethyl ether was added into residual TFA to precipitate the peptide. The peptide precipitate was collected by centrifugation and washed 2 times with diethyl ether (40 ml), then air-dried to give the crude peptide as a pale-yellow solid. The crude PNA was analyzed with LC-MS (Method C) and purified by RP-HPLC method A. The fractions from RP-HPLC were combined and lyophilized for 2 days to give the purified peptides as their corresponding TFA salt. The purity of the peptides was confirmed by LC-MS analysis method D.

### General Peptide-PNA Conjugation

The PNA (0.01 mmol) was dissolved in 3 ml of 10 mM TCEP PBS buffer. In a different vial, the crude peptide (0.012 mmol) was dissolved 2 ml of 10 mM TCEP PBS buffer, and then both solutions were mixed, and pH was adjusted between 7.4 and 8 (pH paper) with 1 M NaOH. The reaction mixture was stirred for 1–4 h and monitored by LC-MS. The crude was purified by RP-HPLC (method B). The fractions from RP-HPLC were analyzed by LC-MS (method C), combined and lyophilized for 2 days to give the purified peptide-PNA conjugates as its corresponding formic acid salt. The purity of the peptides was confirmed by LC-MS analysis method D.

### “One-Pot” Method for the Synthesis of LASP-086

The PNA (LASP-050, 0.005 mmol) was dissolved in 3 ml of 10 mM TCEP PBS buffer. In a different vial, the crude peptide (LASP-078, 0.005 mmol) was dissolved 2 ml of 10 mM TCEP PBS buffer, and then both solutions were mixed, and pH was adjusted between 7.4 and 8 (pH paper) with 1 M NaOH. The progress of the reaction was monitored every hour by LC-MS analysis (method D). Once PNA (LASP-050) was consumed (3 h), the reaction mixture was diluted up to a ∼2 mg/ml concentration (estimated concentration of intermediate), and a solution of 1,4-dibromobutene (1 equivalent) in acetonitrile was added dropwise. The reaction was monitored by LC-MS every hour (4 h). The reaction mixture was purified by RP-HPLC (method B) to obtain LASP-086. The crude was purified by RP-HPLC (method B). The fractions from RP-HPLC were analyzed by LC-MS (method C), combined and lyophilized for 2 days to give the purified peptide-PNA conjugate as its corresponding formic acid salt. The purity of the peptides was confirmed by LC-MS analysis method D.

### Synthesis of LASP-088

The PNA (LASP-050, 0.005 mmol) was dissolved in 3 ml of 10 mM TCEP PBS buffer. In a different vial, the crude peptide (LASP-078, 0.005 mmol) was dissolved 2 ml of 10 mM TCEP PBS buffer, and then both solutions were mixed, and pH was adjusted between 7.4 and 8 (pH paper) with 1 M NaOH. The progress of the reaction was monitored every hour by LC-MS. Once PNA (LASP-050) was consumed (3 h), The reaction mixture was purified by RP-HPLC (method B). The fractions from RP-HPLC were analyzed by LC-MS analysis (method C), combined and lyophilized for 2 days to give the purified peptide-PNA conjugate LASP-085 as its corresponding formic acid salt. The purified LASP-085 was dissolved (0.5 mg/ml) in PBS buffer at pH 7.4, and a solution of DPDS (0.5 equivalent) in acetonitrile was added. The reaction mixture was purified by purification method B to obtain LASP-088. The crude was purified by RP-HPLC (method B). The fractions from RP-HPLC were analyzed by LC-MS analysis (method C), combined, and lyophilized for 2 days to give the purified peptide-PNA conjugate as its corresponding formic acid salt. The purity of the peptides was confirmed by LC-MS analysis method D.

### RP-HPLC Purification Method A

RP-HPLC purification on Shimadzu LC system with a “Prominence” diode array detector (214 nm). A Phenomenex Axia column (Luna C8 (2), 250 × 21.2 mm i.d., 100 Å, 10 μm) was employed with a gradient of 0–60% buffer B over 60 min at a flow rate of 15 ml/min; buffer A was 0.1% TFA/water, and buffer B was 0.1% TFA/acetonitrile.

### RP-HPLC Purification Method B

RP-HPLC purification on a Shimadzu LC system with a “Prominence” diode array detector (214 nm). A Water column (XBridge Peptide BEH C18, 300Å, 150 × 19 mm i.d.) was employed with a gradient of 0–60% buffer B over 60 min at a flow rate of 15 ml/min; buffer A was 0.1% formic acid/water, and buffer B was 0.1% formic acid/acetonitrile.

### LC-MS Analysis Method C

The collected fractions were analyzed by Shimadzu LC-MS -2020 instrument. Solvent A was 0.05% TFA/water, and Solvent B was 0.05% TFA/acetonitrile. A Phenomenex column (Luna C8 (2), 100 × 2.0 mm ID) was used, eluting with a gradient of 0–60% solvent B over 10 min at a flow rate of 0.2 ml/min. Mass spectra were acquired in the positive ion mode with a scan range of 200–2,000 m*/z*.

### LC-MS Analysis Method D

The final purity of peptides, PNA and peptide-PNA conjugates was confirmed by LC-MS. Solvent A was 0.05% TFA/water, and Solvent B was 0.05% TFA/acetonitrile. A Phenomenex column (Luna C8 (2), 100 × 2.0 mm ID) was used, eluting with a gradient of 0–60% solvent B over 30 min at a flow rate of 0.2 ml/min. Mass spectras were acquired in the positive ion mode with a scan range of 200–2,000 m*/z*.

### Antimicrobial Activity

According to the EUCAST guidelines (Eucast, E.C.O.a.S.T., 2021), the inoculum was standardized in sterile saline to the density of a McFarland 0.50 ± 0.02 standard, corresponding to ∼10^8^ CFU/ml of each isolate. The freshly prepared bacterial suspension was evenly inoculated onto agar plates and disks containing 40 μM of peptide-PNA conjugates were applied within 15 min. The diameter of inhibition zones was measured after incubation for 18 h at 37°C. Polymyxin B (40 μM/disk) was employed as the control.

### Membrane Disorganization Measured With Flow Cytometry

Flow cytometry was employed to examine the membrane disorganization in *A. baumannii* 5075R and 5075D following treatments of LASP-072, LASP-097, LASP-132 (0.040 mM, 1, 24 h) (Zhu et al., 2020; Wickremasinghe et al., 2021). Samples were assessed with an ACEA NovoCyte^®^ high-performance benchtop flow cytometer (ACEA Biosciences, Santa Clara, CA, United States) using blue (BL) and violet (VL) laser detection channels. The percentage of bacterial cells with damaged membranes was determined using propidium iodide (PI, Ex/Em 488/660–690 nm).

### *In vitro* Toxicity

Human lung epithelial cells (A549) were obtained from the American Type Culture Collection (ATCC) and maintained in DMEM supplemented with 10% FBS in an incubator containing 5% CO_2_ at 37°C. Cells were plated in 96-well plates at 10^4^ per well for 24 h, then were incubated with LASP-072, LASP-097, LASP-132 (0.04 mM) for another 24 h (Ahmed et al., 2017). The viability (%) of A549 cells was measured with XTT staining (200 μg/ml) combined with phenazine methosulfate (25 μM) in the same culture medium for 2 h at 37°C (De Oliveira Alves et al., 2017). The absorbance was measured at 475 nm using an Infinite M200 plate reader (Tecan group Ltd., Zürich, Switzerland). Cell viability (%) was calculated by the ratio of the background subtracted absorbance of treatments compared with the mean absorbance of untreated controls (Riss et al., 2016).

## Results and Discussion

**FIGURE 2:**
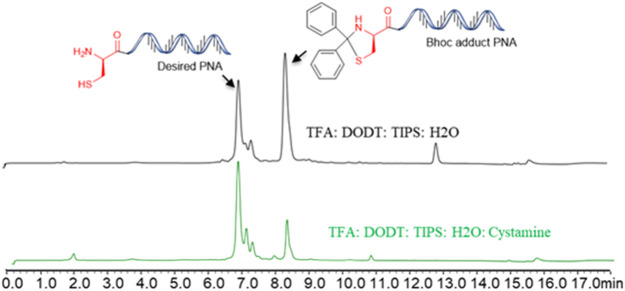
The benzhydryloxyearbonyl (Bhoc) adduct during the Trifluoroacetic acid (TFA) cleavage of PNA and optimisation of cleavage protocol.

All PNAs were synthesized using Fmoc/Bhoc protected PNA building blocks by standard solid-phase synthesis (SPPS) protocol (Table 1). In our initial attempt to cleave the *N*-terminal cysteine containing PNA (Cys-PNA) from rink amide resin, we observed the formation of a Bhoc-adduct as a major product (characterized by a 166 Dalton higher mass), resulting in a significant loss in the yield of the desired PNA (Figure 2, purified yield only 5%) (Goodwin et al., 1998). The benzhydryloxycarbonyl (Bhoc) protecting group generates “benzhydryl cation” during the trifluoroacetic acid (TFA) cleavage. This benzhydryl cation reacts with the 1,2- aminothiol moiety of *N*-terminal cysteine and produces a Bhoc-adduct. Previously, Goodwin et al. (1998) utilized the *S*-t-butylmereapto group as an orthogonal protecting group strategy to synthesize Cys-PNAs. The use of *S*-t-butylmereapto protected *N*-terminal cysteine residue prevented the Bhoc adduct formation. However, additional deprotection and purification steps were needed to obtain the desired Cys-PNA. Notably, the reported strategy is time-consuming and may present a bottleneck for structure-function studies involving large peptide-PNA libraries. Therefore, we optimized the cleavage conditions that improved the Cys-PNA yields with minimum steps. Initially, we investigated four cleavage solutions, each with different scavengers (4-Methoxythiophenol, 4-Mercaptophenol, Cysteine, and Cysteamine) that preferentially react with “benzhydryl cation”. The LC-MS analysis suggested that cysteamine exhibited a better ability to scavenge the “benzhydryl cation” (Supplementary Figure S2). We then optimized the concentration of cysteamine and revealed that 2 equivalents of cysteamine per Bhoc group was essential to avoid the formation of the Bhoc adduct. The optimized cleavage conditions significantly improved the yields of the Cys-PNA (LASP-050, 55%).

**FIGURE 3:**
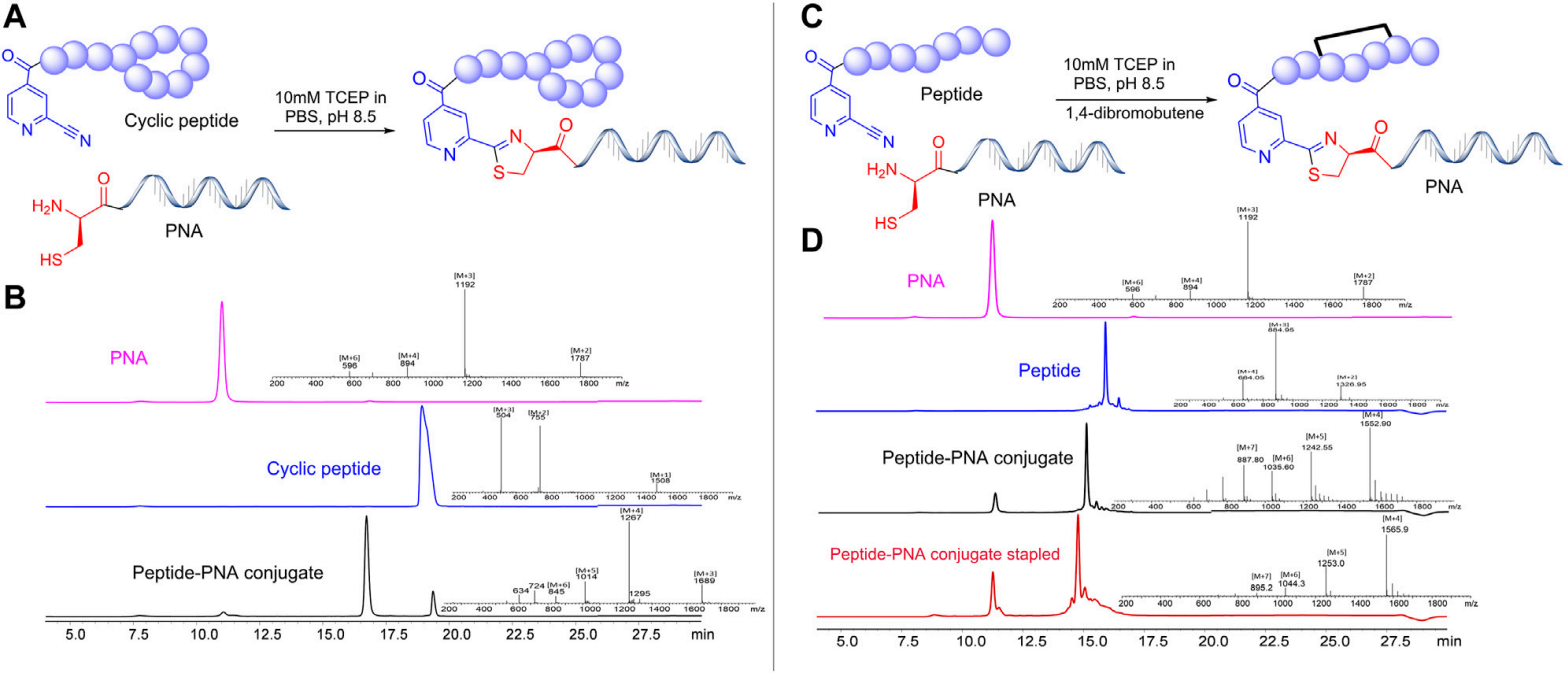
Synthetic strategies and efficiency of Cys-CINA in the synthesis of complex peptide-PNA conjugates. **(A)** Synthesis of cyclic peptide-PNA conjugate: CINA-OctGly-Dab-Thr-Dab-Dab*-Dab-D-Phe-Leu-Dab-Dab-Thr* (1. 2 equivalents), PNA: Cys-PEG-Cyt-Thy-Cyt-Ade-Thy-Ade-Cyt-Thy-Cyt-Thy-Thy-Gua-Lys-NH2 (1 equivalent), 10 M TCEP, PBS, pH 7.4. **(B)** Analytical HPLC spectra for polymyxin, PNA and crude polymyxin-PNA conjugate. **(C)** “One-pot” synthesis of thioether cross-linked cathelicidin-PNA conjugate: CINA-PEG-Val-Cys-Lys-Arg-Trp-Lys-Lys-Trp-Lys-Arg-Lys-Trp-Lys-Lys-Trp-Cys-Val-NH2 (1. 2 equivalents), PNA: Cys-PEG-Cyt-Thy-Cyt-Ade-Thy-Ade-Cyt-Thy-Cyt-Thy-Thy-Gua-Lys-NH2 (1 equivalent) 10 M TCEP, PBS, pH 7.4, 1,2-dibromobutene. **(D)** Analytical HPLC spectra for linear cathelicidin, PNA, crude cathelicidin-PNA conjugate, and *in situ* side-chain cross-linking. [TCEP: tris(2-carboxyethyl) phosphine, PEG: 8-Amino-3,6-Dioxaoctanoic Acid, PBS: phosphate buffer saline].

Once a large scale Cys*-*PNA (LASP-050, Supplementary Figure S1) was accomplished, we synthesized 5 structurally diverse peptides with the CINA moiety using standard SPPS protocol. The synthesis of cyclic peptide-PNA conjugates involves critical steps such as handling orthogonally protected cyclic peptides with labor-intensive synthesis and multiple purifications (Alam et al., 2011; Shinbara et al., 2020). Thus, the synthesis of cyclic peptide-PNA conjugates presents significant challenges. To investigate the efficiency of CINA-conjugation, we synthesized a polymyxin analogue (LASP-026, Table 1, Supplementary Figure S3) with a CINA moiety. The presence of *N-*terminal CINA moiety did not affect the yields of polymyxin analogue. The CINA-peptide LASP-026 and Cys-PNA LASP-050 (Figure 3A) were then used to optimize conjugation conditions. We observed that the Cys-PNA (LASP-50) was completely conjugated to the polymyxin peptide (LASP-026) within 2 h (Figure 3B). The use of aqueous buffers for the conjugation reaction enabled direct purification. Since the thiazolidine ring is not stable in 0.1% TFA buffers (Morewood and Nitsche, 2021), the peptide-PNA conjugate (LASP-059) was purified using a less acidic formic acid buffer (Nitsche et al., 2019). The optimized Cys-CINA conjugation method was applied to synthesize all other peptide-PNA conjugates (Table 1). Subsequently, we investigated the effect of the position of the CINA group and peptide length on the efficiency of conjugation reaction using LASP-054 and LASP-077 (Table 1, Supplementary Figures S4, S5). A lysine-side-chain anchored CINA peptide RXR (Popella et al., 2021) and an antimicrobial peptide “drosocin” containing 19 amino acids were selected as templates (Hansen et al., 2016). The conjugation reactions were completed within 4 h, and both peptide-PNA conjugates, LASP-058 (95%) and LASP-084 (90%), were obtained with high yields. The CINA group is highly selective towards *N*-terminal cysteine and enables the orthogonal disulfide bond formation or thioether cross-linking with intra-chain free thiols. We explored this selectivity of Cys-CINA chemistry and synthesized the disulfide-linked cathelicidin-PNA conjugate (LASP-088) in two steps. Interestingly, the conjugation buffer contains TCEP as a reducing agent, which provides an opportunity to explore the facile formation of thioether bonds using halogenated cross-linking agents such as 1,4-dibromobutene (Heinis et al., 2009; Fadzen et al., 2017). Hence, we utilized this key attribute of the Cys-CINA approach and developed a “one-pot” protocol for the conjugation and side-chain cross-linking. A cathelicidin analogue was an ideal template for investigating this strategy (Mwangi et al., 2019). The linear peptide (LASP-078, Supplementary Figure S6) was quantitatively converted to conjugate intermediate within 3 h (Figure 3C). Excitingly, *in situ* formation of thioether linkage between 1,4-dibromobutene and the free thiols of non-terminal cysteines yielded LASP-086 as a major product (Figure 3D). Notably, Cys-CINA conjugation reactions were efficiently conducted in an aqueous buffer, which allowed facile handling and purification of the conjugates.

**FIGURE 4:**
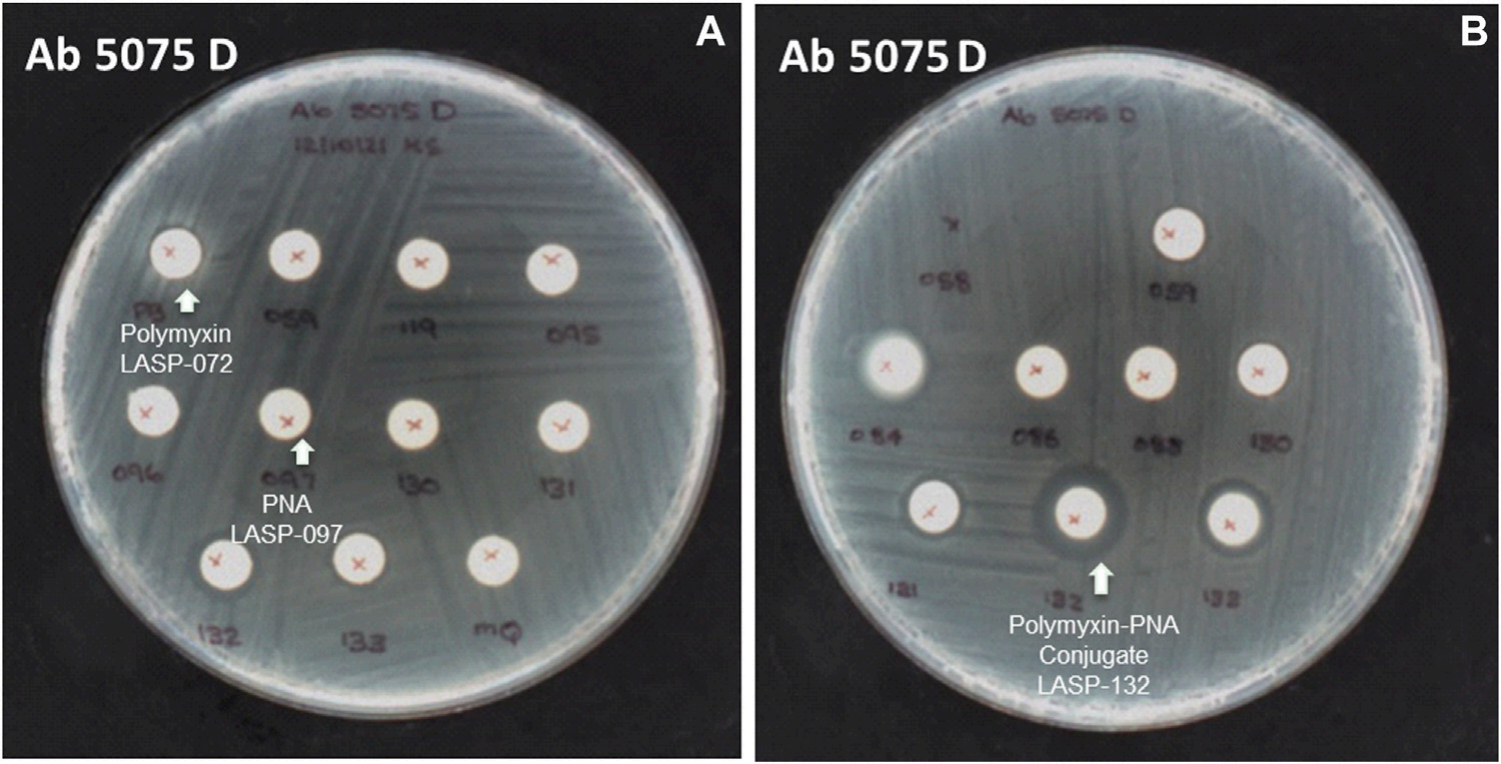
The inhibition zone of peptides, PNA, and peptide-PNA conjugates against *A. baumannii* 5075D **(A)** polymyxin and PNA No zones of inhibition observed, **(B)** polymyxin-acpP PNA conjugate zone of inhibition observed.

The selected peptide sequences are known for their antimicrobial activity (Le et al., 2017). Therefore, we evaluated the antimicrobial activity of synthesized peptide-PNA conjugates using the Disc Diffusion Assay (DDA) against a panel of MDR Gram-negative bacteria consisting of *P. aeruginosa*, *A. baumannii*, *K. pneumoniae*, *E. coli*, including the isolates highly polymyxin-resistant *A. baumannii* 5075R, and *A. baumannii* 5075D (Figure 4 and Supplementary Table S1). No zone of bacterial growth inhibition was observed in the presence of conjugates LASP-058, LASP-086, and controls (unconjugated peptide and PNAs). However, conjugates LASP-084 and LASP-088 showed a zone of inhibition (diameter of 8 mm) against *E. coli* DH5α, *A. baumannii* 5075, and *A. baumannii* 5075D. Interestingly, *A. baumannii* 5075, which exhibits a polymyxin-dependent resistance phenotype, showed greater bacterial growth in the presence of LASP-084. The polymyxin-PNA conjugate LASP-059, showed better antimicrobial activity compared to LASP-058, 084, 088, with the diameters of inhibition zones ranging between 7 and 10 mm (Supplementary Table S1). These experiments suggested that polymyxin peptides can be an effective carrier for antimicrobial PNA. Therefore, we further synthesized 10-mer PNAs targeting four bacterial essential genes *anti-rpo*A LASP-095 (Abushahba et al., 2016), *anti-murA* LASP-096 (Goh et al., 2009; Mondhe et al., 2014), *anti-acpP* LASP-097 (Hansen et al., 2016), and *anti-rpoD* LASP-119 (Bai et al., 2012; Alajlouni and Seleem, 2013). During the synthesis of the next set of peptide-PNA conjugates, we switched the direction of the conjugation handles to investigate the impact on the efficiency of conjugation. Here, PNAs were synthesized with an *N*-terminal CINA moiety (LASP-095, 096, 097 and 119, Table 1) and a polymyxin analogue (LASP-072, Table 1) was synthesized containing an *N*-terminal cysteine residue (Supplementary Figure S3). Pleasingly, the alteration of the conjugating handle did not affect the efficiency of the Cys-CINA conjugation (90–98%, Table 1). The synthesized peptide-PNA conjugates LASP-130, -131, -132 and -133 showed zones of inhibition in bacterial growth (diameter in the range of 11–14 mm). Importantly, all conjugates (LASP-130 to 133, Supplementary Table S1, Supplementary Figures S7–S13) showed antimicrobial activity against *A. baumannii* 5075R and *A. baumannii* 5075D without any observed dependent phenotype (Figure 4). Excitingly, LASP-132 showed a maximum zone of inhibition, indicating the potential promise of polymyxin *anti-acpP* PNA conjugates for the development of new antimicrobial agents.

**FIGURE 5:**
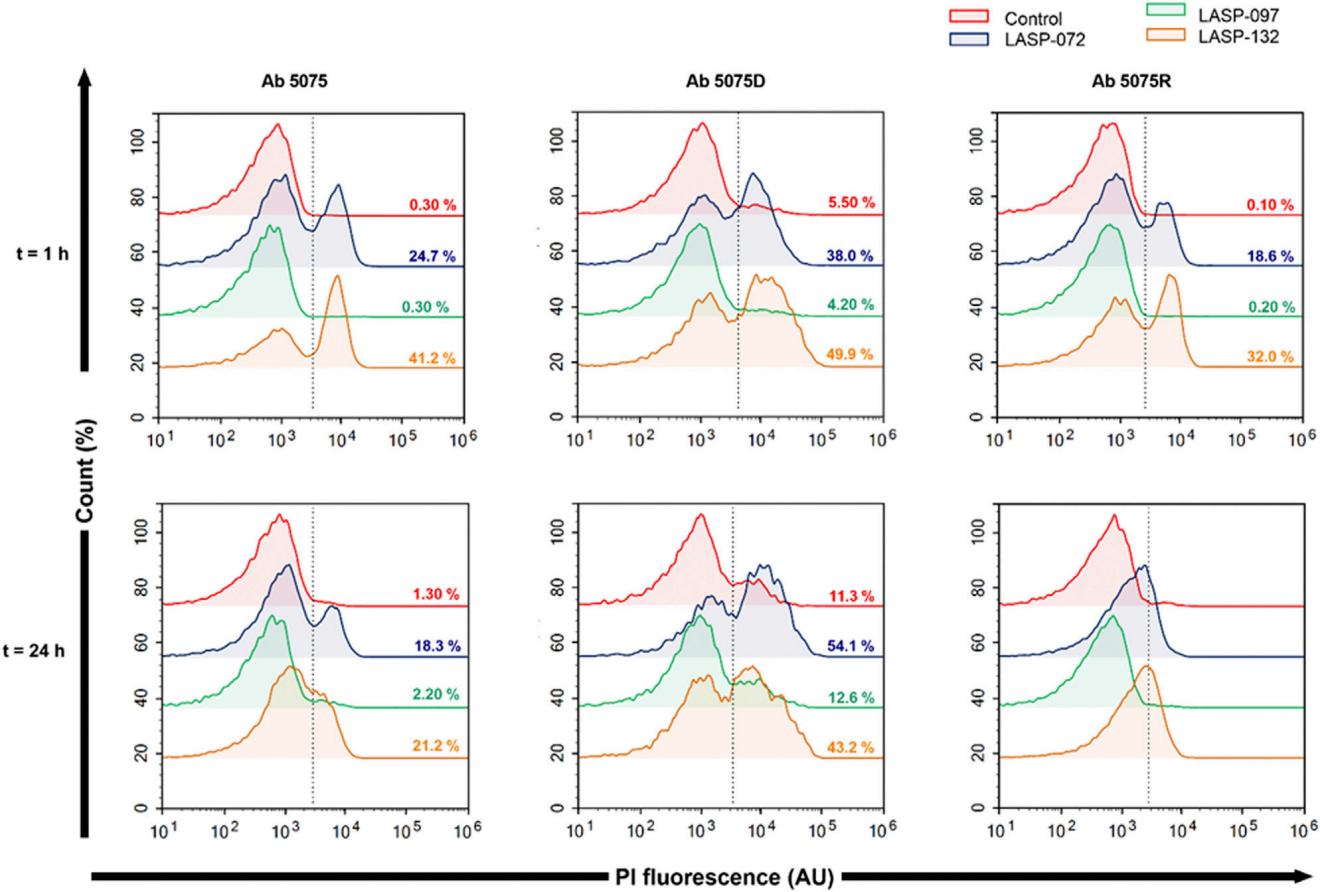
Bacterial flow cytometry analysis. Early log phase of *A. baumannii* 5075, 5075D and 5075R were treated with LASP-072, LASP-097 and LASP-132 (0.04 mM, 1 and 24 h). Bacterial cells were stained with PI and analyzed by fluorescence-activated cell sorting (FACS) flow cytometry. The histograms represent the percentage of PI-positive cells after each treatment.

**FIGURE 6:**
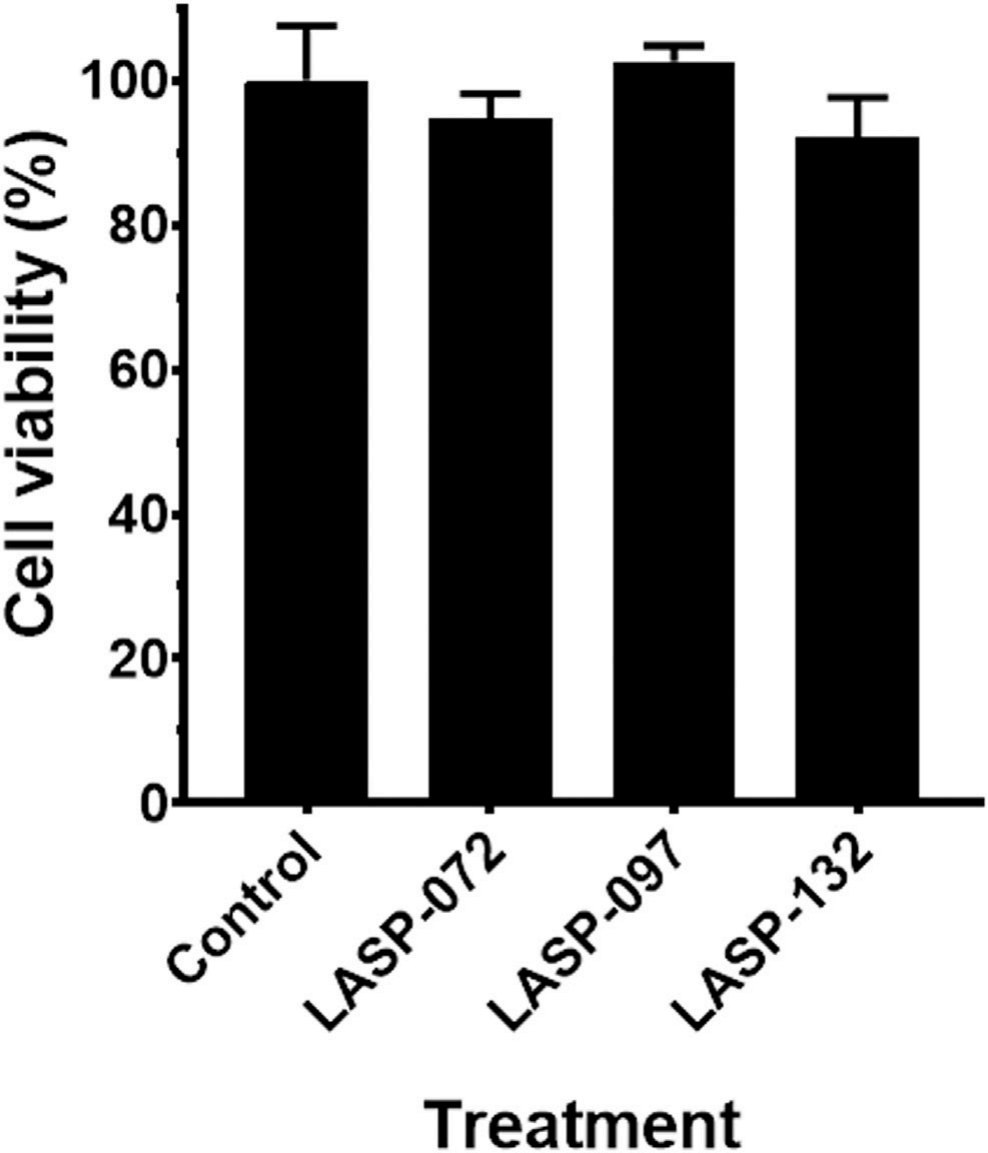
Cell viability (%) of the A549 cells treated with LASP-072, LASP-097 and LASP-132 (0.04 mM, 24 h) using XTT assay (mean ± SD; *n* = 4). Tukey’s multiple comparisons test was performed and *p* > 0.05.

Intrigued by the activity of LASP-132 against polymyxin-resistant *A. baumannii* isolates (5075R with LPS phosphoethanolamine modification, and LPS loss polymyxin-dependent resistant isolate 5075D), we investigated the potential mode of action of our PNA conjugates using flow cytometry (Figure 5). The increase in proportions of PI-positive cells (%) was observed following the treatment with LASP-072 and LASP-132 for all the isolates tested at 1 h; whereas these proportions were minimal in untreated controls at the same time-point. Our results show the membrane damage in polymyxin-resistant *A. baumannii* 5075R and 5075D by LASP-072 and LASP-132, indicating re-sensitization of the polymyxin-resistant bacteria to PNAs. Our finding also highlights the outer membrane as a potential target for LASP-132 besides its intracellular antisense activity (Bergen et al., 2006; Rasmussen et al., 2007). Importantly, the proportions of PI-positive bacterial cells (%) after the treatment of LASP-132 was higher (11.9%–16.5%) compared to that by LASP-072 at 1 h, indicating a higher capacity of membrane disorganization activity of the former. The membrane damage activity of LASP-132 is well correlated with the antimicrobial activity, where a larger zone of inhibition was observed for LASP-132 compared to that of LASP-072 (Figure 4). Interestingly, membrane damage activity of LASP-132 was decreased at 24 h; in particular, no distinct population of PI-positive cells was observed in *A. baumannii* 5075R, possibly due to the remodelling of the bacterial outer membrane at 24 h (Zhu et al., 2020). We also investigated the *in vitro* toxicity of LASP-072, LASP-097, and LASP-132 in human lung epithelial A549 cells (Figure 6) (Ahmed et al., 2017; Mea et al., 2020). Interestingly, the antibacterial LASP-072 and LASP-132 caused <10% death of A549 cells and no significant difference was observed with the untreated control samples. Our cell culture results indicate the relatively low toxicity of both compounds. Overall, our findings suggest that LASP-132 is a promising hit for further development of PNA-peptide conjugates against MDR *A. baumannii*, including those resistant to the last-line polymyxins.

## Conclusion

We successfully developed an efficient and biocompatible strategy to conjugate complex peptides with PNAs using the 2-cyanoisonicotinamide moiety. This method utilizes economic and commercially available 2-cyanoisonicotinic acid that can be directly introduced to peptide and PNA by SPPS. We also developed a new cleavage protocol to enhance the yields of *N*-terminal cysteine containing PNAs. Importantly, the compatibility of Cys-CINA conjugation with chemically diverse peptides, including cyclic and cysteine-rich peptides, allows scalable and cost-effective syntheses of complex peptide-PNA analogues. Further, we developed the “one-pot” conjugation and side-chain cross-linking protocol for the cysteine-rich peptide. The Cys-CINA conjugation strategy offers a versatile, high yielding and efficient approach for synthesizing peptide-PNA conjugates that will advance the current oligonucleotide-based chemical biology research. Finally, this study identified a polymyxin-PNA conjugate (LASP-132) as a promising antimicrobial agent against MDR *A. baumannii*. The LASP-132 serves as a template for the future development of peptide-PNA antibiotics underway in our laboratory.

## Data Availability

The original contributions presented in the study are included in the article/Supplementary Material, further inquiries can be directed to the corresponding author.
