# Supplementary material for: An Efficient Approach for the Design and Synthesis of Antimicrobial Peptide-Peptide Nucleic Acid Conjugates
Source: Front Chem. 2022 Mar 15;10:843163. doi: 10.3389/fchem.2022.843163 (PMC8964499; doi:10.3389/fchem.2022.843163)
Supplement: Supplementary file 1 [file DataSheet1.PDF]

An efficient approach for the design and synthesis of antimicrobial peptide-peptide nucleic acid conjugates

Nitin A. Patil[1]*, Varsha J Thombare[2], Rong Li[1], Xiaoji He[1], Jing Lu[1, 2], Heidi H. Yu[1], Hasini Wickremasinghe[1], Tony Velkov[2], Kade D. Roberts[1], Jian Li[1]

[1]Biomedicine Discovery Institute, Infection & Immunity Program and Department of Microbiology, Monash University, Melbourne, Australia.

[2]Department of Biochemistry and Pharmacology, The University of Melbourne, Melbourne, VIC 3010, Australia.

* Corresponding authors:

Dr Nitin Patil at 15 Innovation Walk, Monash University, Clayton Campus, Melbourne 3800, Australia. Telephone: +61 3 99051015. Email: nitin.patil@monash.edu

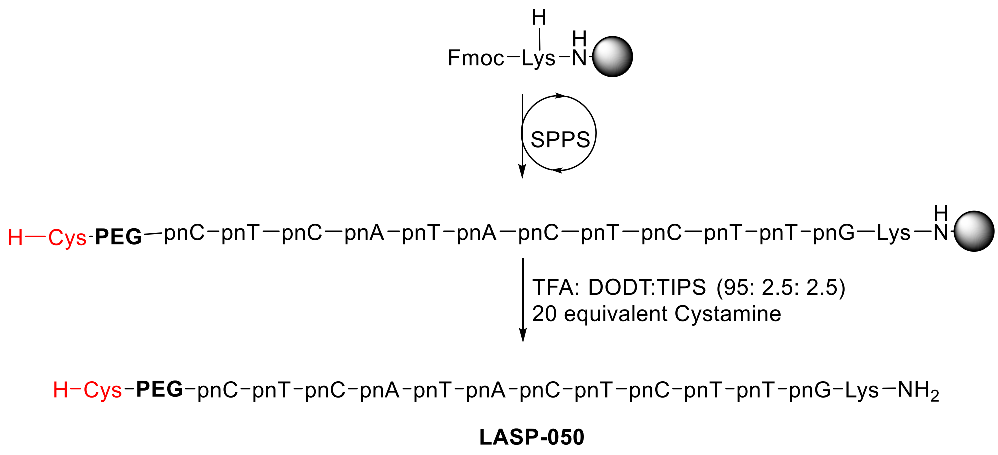

Figure S1: Synthesise of PNA with *N*-terminal cysteine residue

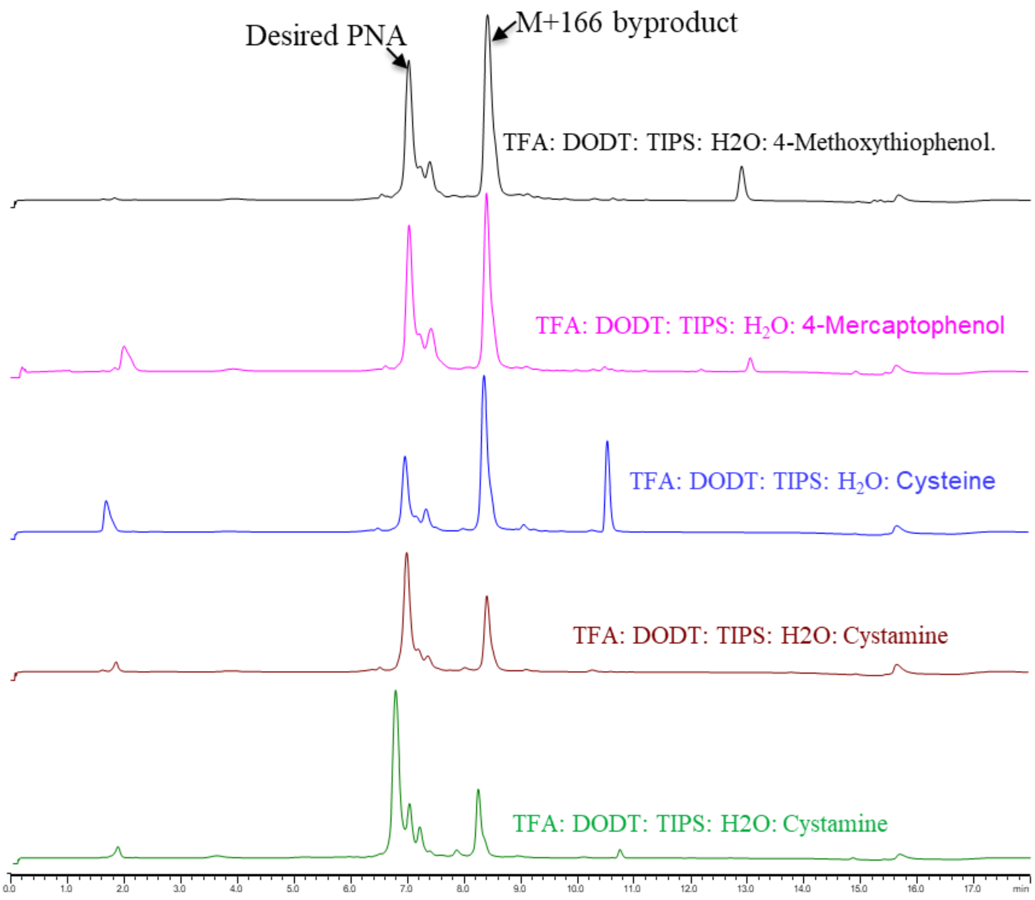

Figure S2. Reaction kinetic study of Cys-PNA (LASP-050) with different cleavage scavengers.

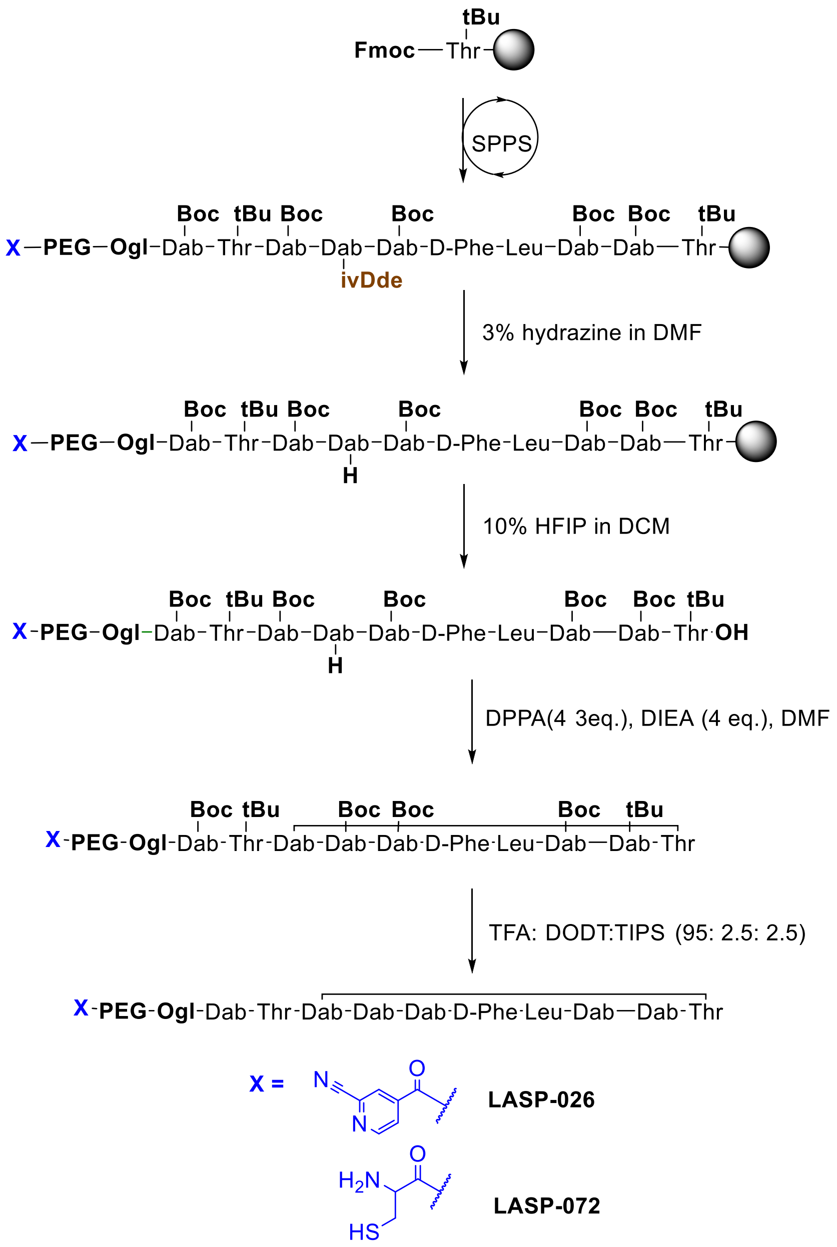

Figure S3: Synthesise of polymyxin analogue LASP-026, which contains a CINA handle and LASP-072 with *N*-terminal Cysteine residue.

Synthesis of polymyxin analogues: The linear peptide sequence was built using the general peptide synthesis protocol reported in the methods section. Removal of ivDde protecting group and protected peptide cleavage was performed on a Protein Technologies Prelude automated peptide synthesiser. Removal of the ivDde protecting group was achieved with 3% hydrazine in DMF (4 × 15 min). The protected linear peptide was then cleaved from the resin by treating

the resin with 10% hexafluoroisopropanol (HFIP) in DCM (1 × 30 min, 1 × 5 min). This solution was concentrated in vacuo to give the crude protected linear peptide. The protected linear peptide was dissolved in DMF (5 mL) to which DIPEA 0.6 mmol, 104 μL (6 molar equivalents relative to the loading of the resin) and DPPA, 0.3 mmol, 0.65 μL (3 molar equivalents relative to the loading of the resin) were added. The solution was stirred overnight at room temperature. The reaction solution was then concentrated under vacuum for a minimum 6 h to give the crude protected cyclic peptide. The resulting residue was taken up in a solution of 2.5% DODT, 5% TIPS in TFA and stirred at room temperature for 90 minutes. To this solution, 40 mL of diethyl ether was added. The resulting precipitate was collected by centrifugation and washed twice with diethyl ether (40 mL), then air-dried in a fume hood to give the crude cyclic peptide as a pale-yellow solid. The resulting solid was taken up in Milli-Q water (5 mL) and de-salted using a Vari-Pure IPE SAX column. The crude cyclic lipopeptide was then subjected to RP-HPLC purification.

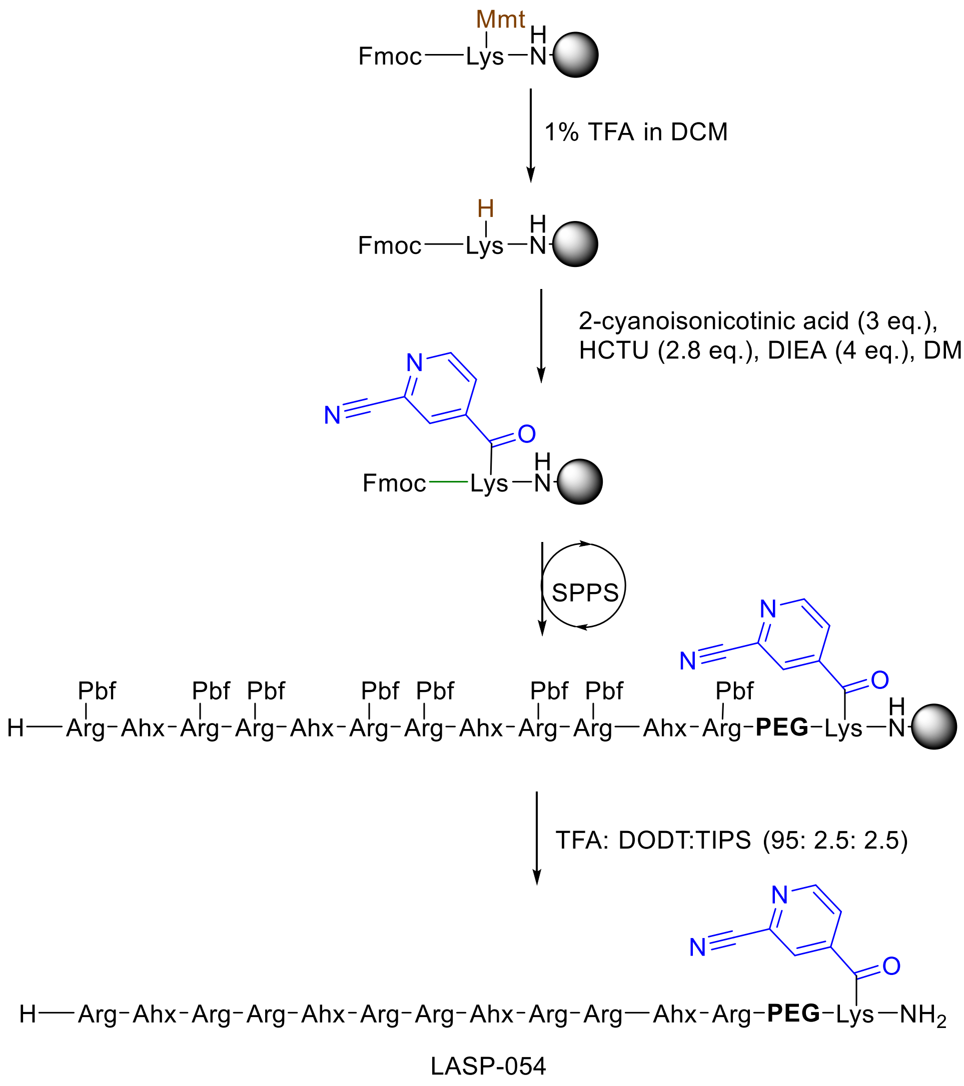

Figure S4: Synthesise of peptide RXR with a side-chain CINA handle

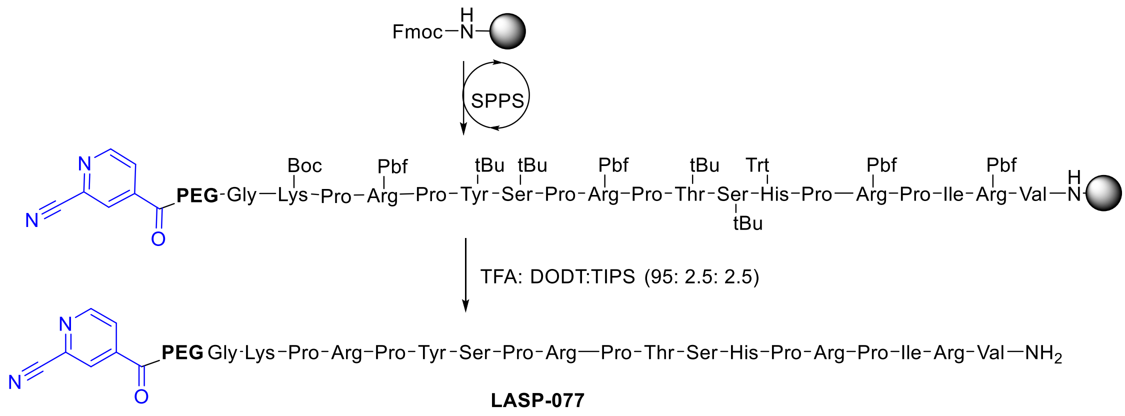

Figure S5: Synthesise of linear drosocin

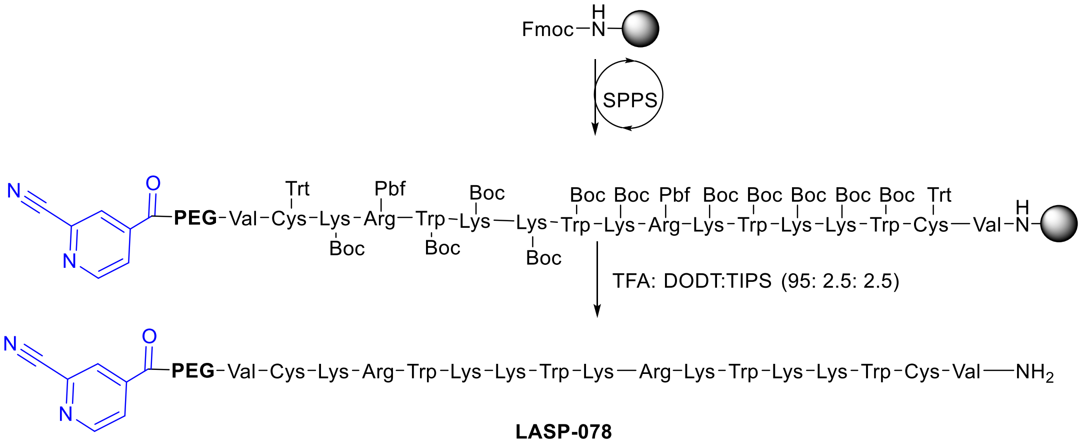

LASP-078

Figure S6: Synthesise of linear cathelicidin

Table S1: Inhibition zone (mm) for peptide, PNA, and peptide-PNA conjugates

	Zone of inhibition diameter (mm)						
Volume correction per disk based on Mw equivalent to 40 ug of LASP-072	Pa ATCC 27853	Ab ATCC 17978	Kp ATCC 700721	E. coli DH5α	Ab 5075	Ab 5075 R	Ab 5075 D
PNA analogues							
LASP-058	0	0	0	0	0	0	0
LASP-095	0	0	0	0	0	0	0
LASP-096	0	0	0	0	0	0	0
LASP-097	0	0	0	0	0	0	0
LASP-119	0	0	0	0	0	0	0
Peptide-PNA analogues							
LASP-059	7	8	8	10	9	0	7
LASP-084	0	0	0	8	0	0	7
LASP-086	0	0	0	0	0	0	0
LASP-088	0	0	0	8	7	0	0
LASP-130	9	9	9	12	10	7	0
LASP-131	8	10	8	11	10	7	8
LASP-132	13	12	12	14	12	11	12
LASP-133	10	11	10	12	11	8	9
Peptide analogues							
LASP-026	14	13	13	15	13	11	9
LASP-054	0	9	8	9	8	8	9
LASP-072	14	13	14	17	14	13	9
LASP-077	0	0	0	0	0	0	0
LASP-078	0	0	0	0	0	0	0
LASP-087	0	8	7	13	8	0	0

Supplementary Methods

PNAs

LASP-050:

H·Cys–**PEG**–Thy–Gau–Cyt–Thy–Cyt–Ade–Thy–Ade–Cyt–Thy–Cyt–NH_2

LASP-050 was purified by method A and analyzed by method D. TFA salt was obtained in a yield of 136 mg, retention time (t_R) at 214 nm = 11.007 min (purity: 98.93 %). ESI-MS analysis of peak at 11.00 min: *m/z*, $[M+2H]^{2+}$ 1787, $[M+3H]^{3+}$ 1192, $[M+4H]^{4+}$ 894, $[M+5H]^{5+}$ 596. Calculated mass (monoisotopic) for LASP-050 ($C_{143}H_{192}N_{66}O_{44}S$) 3569.45

LASP-095:

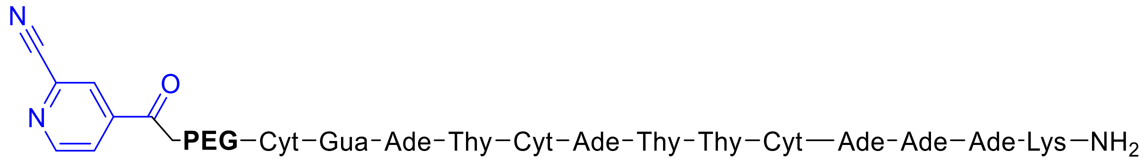

LASP-095 was purified by method A and analyzed by method D. TFA salt was obtained in a yield of 56 mg, retention time (t_R) at 214 nm = 12.417 min (purity: 87.96 %). ESI-MS analysis of peak at 12.41 min: *m/z*, $[M+2H]^{2+}$ 1821.8, $[M+3H]^{3+}$ 1214.8, $[M+4H]^{4+}$ 911.35, $[M+5H]^{5+}$ 729.30. Calculated mass (monoisotopic) for LASP-095 ($C_{148}H_{187}N_{75}O_{39}$) 3638.50

LASP-096:

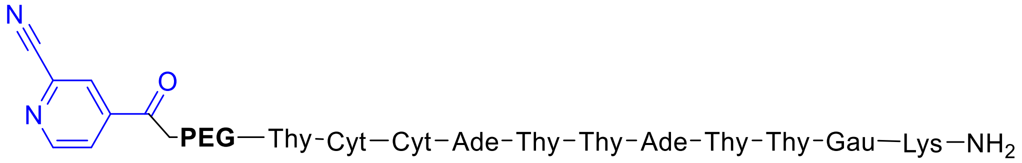

LASP-096 was purified by method A and analyzed by method D. TFA salt was obtained in a yield of 40 mg, retention time (t_R) at 214 nm = 13.248 min (purity: 96.18 %). ESI-MS analysis of peak at 13.24 min: *m/z*, $[M+2H]^{2+}$ 1549.40, $[M+3H]^{3+}$ 1033.20, $[M+4H]^{4+}$ 775.15. Calculated mass (monoisotopic) LASP-096 ($C_{127}H_{163}N_{57}O_{38}$) 3096.06

LASP-097:

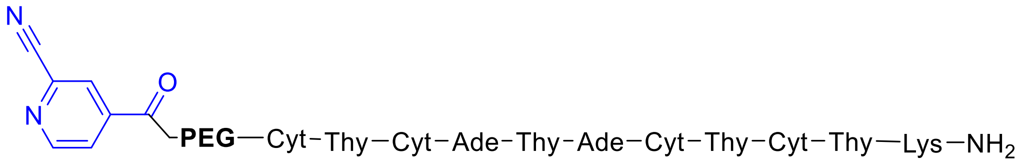

LASP-097 was purified by method A and analyzed by method D. TFA salt was obtained in a yield of 47 mg, retention time (t_R) at 214 nm = 12.671 min (purity: 97.67 %). ESI-MS analysis of peak at 12.67 min: *m/z*, $[M+2H]^{2+}$ 1521.90, $[M+3H]^{3+}$ 1014.85, $[M+4H]^{4+}$ 761.45. Calculated mass (monoisotopic) for LASP-097 ($C_{125}H_{162}N_{56}O_{37}$) 3041.03.

LASP-119:

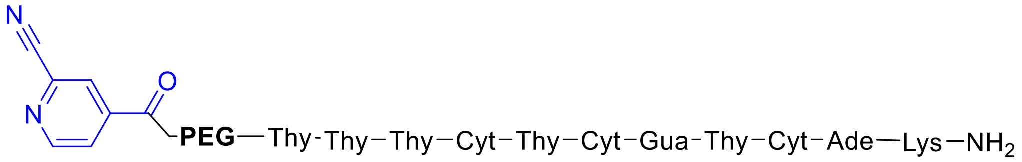

LASP-119 was purified by method A and analyzed by method D. TFA salt was obtained in a yield of 23 mg, retention time (t_R) at 214 nm = 13.637 min (purity: 98.58 %). ESI-MS analysis of peak at 13.63 min: *m/z*, $[M+2H]^{2+}$ 1537.45, $[M+3H]^{3+}$ 1025.25, $[M+4H]^{4+}$ 769.25. Calculated mass (monoisotopic) for LASP-119 ($C_{126}H_{163}N_{55}O_{39}$) 3073.88.

Peptides

LASP-026:

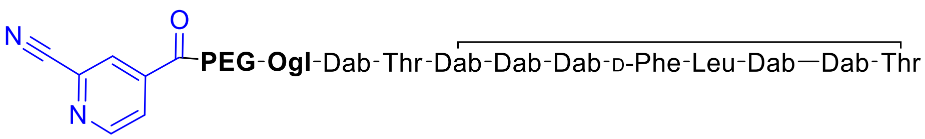

LASP-026 was purified by method A and analyzed by method C. TFA salt was obtained in a yield of 63 mg, retention time (t_R) at 214 nm = 18.919 min (purity: 96.51 %). ESI-MS analysis of peak at 18.91 min: *m/z* (monoisotopic) $[M+H]^{+}$ 1606.25, $[M+2H]^{2+}$ 803.50, $[M+3H]^{3+}$ 536.10. Calculated mass (monoisotopic) LASP-026 ($C_{70}H_{114}N_{20}O_{17}$) 1605.88.

LASP-054

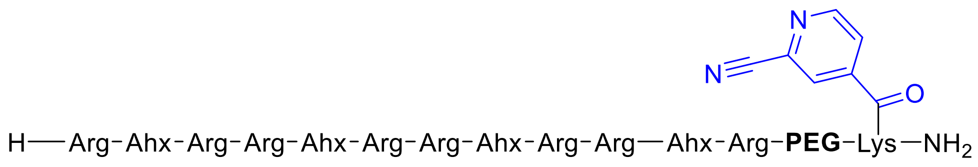

LASP-054 was purified by method A and analyzed by method C. TFA salt was obtained in a yield of 100 mg, retention time (t_R) at 214 nm = 11.538 min (purity: 92.85 %). ESI-MS analysis of peak at 11.53: *m/z* (monoisotopic) $[M+2H]^{2+}$ 1062.45, $[M+3H]^{3+}$ 708.75, $[M+4H]^{4+}$ 531.85. Calculated mass (monoisotopic) LASP-054 ($C_{91}H_{168}N_{42}O_{17}$) 2121.36

LASP-077

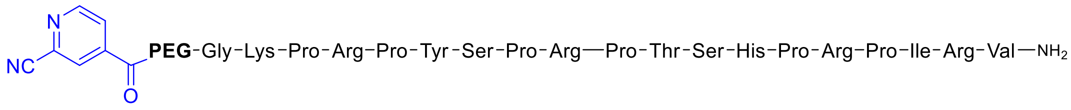

LASP-077 was purified by method A and analyzed by method C. TFA salt was obtained in a yield of 43 mg, retention time (t_R) at 214 nm = 10.813 min (purity: 95.46 %). ESI-MS analysis of peak at 10.81 min: *m/z* (monoisotopic) $[M+2H]^{2+}$ 1237.75, $[M+3H]^{3+}$ 825.55, $[M+4H]^{4+}$ 619.45. Calculated mass (monoisotopic) for LASP-077 ($C_{111}H_{174}N_{38}O_{27}$) 2472.3 .

LASP-078

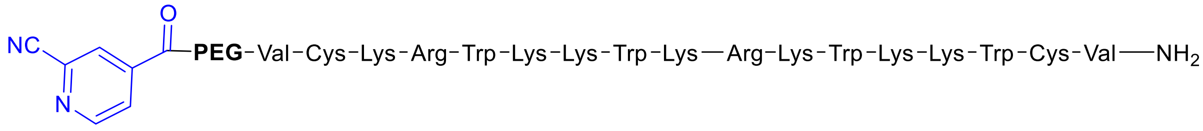

LASP-078 was purified by method A and analyzed by method C.TFA salt was obtained in a yield of 50 mg, retention time (t_R) at 214 nm = 16.051 min (purity: 96.20 %). ESI-MS analysis of peak at 16.05 min: *m/z* (monoisotopic) $[M+2H]^{2+}$ 1326.95, $[M+3H]^{3+}$ 884.95, $[M+4H]^{4+}$ 664.05. Calculated mass (monoisotopic) for LASP-078 ($C_{127}H_{192}N_{38}O_{21}S_2$) 2651.30

LASP-072

Cys-**PEG**-**Ogl**-Dab-Thr-Dab-Dab-Dab-D-Phe-Leu-Dab—Dab-Thr

LASP-072 was purified by method A and analyzed by method C. TFA salt was obtained in a yield of 126 mg, retention time (t_R) at 214 nm = 12.085 min (purity: 95.05 %). ESI-MS analysis of peak at 12.08 min: *m/z* (monoisotopic) $[M+H]^+$ 1481.30, $[M+2H]^{2+}$ 741.50, $[M+3H]^{3+}$ 494.75. Calculated mass (monoisotopic) for LASP-072 ($C_{66}H_{116}N_{19}O_{17}S_1$) 1480.88.

Peptide PNA conjugates

LASP-058

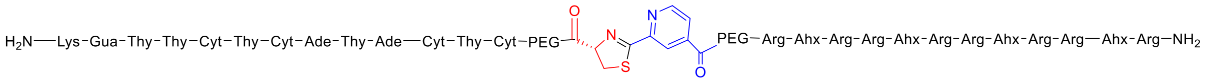

LASP-058 was purified by method B and analyzed by method C. Formic acid salt was obtained in a yield of 5.4 mg, retention time (t_R) at 214 nm = 12.468 min (purity: 95.01 %). ESI-MS analysis of peak at 12.46 min: *m/z*, $[M+4H]^{4+}$ 1420.9, $[M+5H]^{5+}$ 1136.8, $[M+7H]^{7+}$ 710.8. Calculated mass (monoisotopic) for LASP-058 ($C_{234}H_{356}N_{107}O_{61}S$) 5676.10.

LASP-059

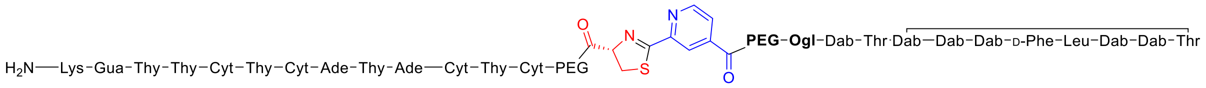

LASP-059 was purified by method B and analyzed by method C. Formic acid salt was obtained in a yield of 19 mg, retention time (t_R) at 214 nm = 19.73 min (purity: 96.94 %). ESI-MS analysis of peak at 19.73 min: *m/z*, $[M+3H]^{3+}$ 1688.9, $[M+4H]^{4+}$ 1266.9, $[M+5H]^{5+}$ 1013.6. Calculated mass (monoisotopic) for LASP-059 ($C_{213}H_{302}N_{85}O_{61}S$) 5061.3.

LASP-084

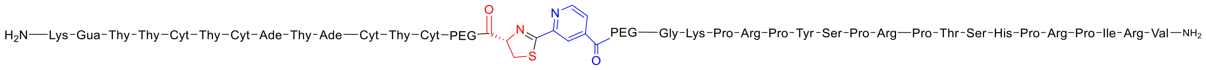

LASP-084 was purified by method B and analyzed by method C. Formic acid salt was obtained in a yield of 3.4 mg, retention time (t_R) at 214 nm = 13.302 min (purity: 97.12 %). ESI-MS analysis of peak at 13.30 min: *m/z*, [$[M+4H]^{4+}$ 1508.4, $[M+5H]^{5+}$ 1206.4. Calculated mass (monoisotopic) for LASP-084 ($C_{254}H_{362}N_{103}O_{71}S$) 6026.3

LASP-085

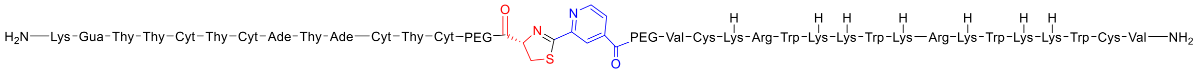

LASP-085 was purified by method B and analyzed by method C. Formic acid salt was obtained, retention time (t_R) at 214 nm = 19.312 min (purity: 83.45 %). ESI-MS analysis of peak at 19.31 min: *m/z*, [$[M+4H]^{4+}$ 1552.90, $[M+5H]^{5+}$ 1242.55, $[M+6H]^{6+}$ 1035.6, , $[M+7H]^{7+}$ 887.80, $[M+8H]^{8+}$ 777.0 Calculated mass (monoisotopic) for LASP-088 ($C_{270}H_{390}N_{103}O_{65}S_3$) 6205.73.

LASP-086

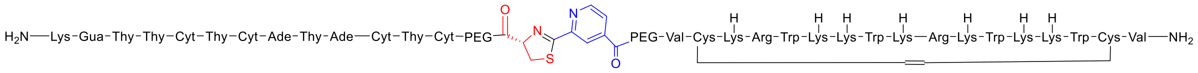

LASP-086 was purified by method B and analyzed by method C. Formic acid salt was obtained in a yield of 136 mg, retention time (t_R) at 214 nm = 18.467 min (purity: 92.24 %). ESI-MS analysis of peak at 18.46 min: *m/z*, $[M+4H]^{4+}$ 1565.9, $[M+5H]^{5+}$ 1253.0, $[M+6H]^{6+}$ 1044.3 . Calculated mass (monoisotopic) for LASP-086 ($C_{274}H_{396}N_{103}O_{65}S_3$) 6258.84.

LASP-088

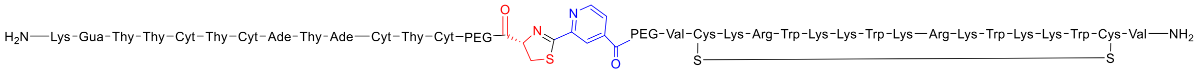

LASP-088 was purified by method B and analyzed by method C. Formic acid salt was obtained in a yield of 13.1 mg, retention time (t_R) at 214 nm = 18.275 min (purity: 96.30 %). ESI-MS analysis of peak at 18.27 min: *m/z*, $[M+4H]^{4+}$ 1552.4, $[M+5H]^{5+}$ 1242.1, $[M+6H]^{6+}$ 1035.3. Calculated mass (monoisotopic) for LASP-088 ($C_{270}H_{388}N_{103}O_{65}S_3$) 6202.73.

LASP-130

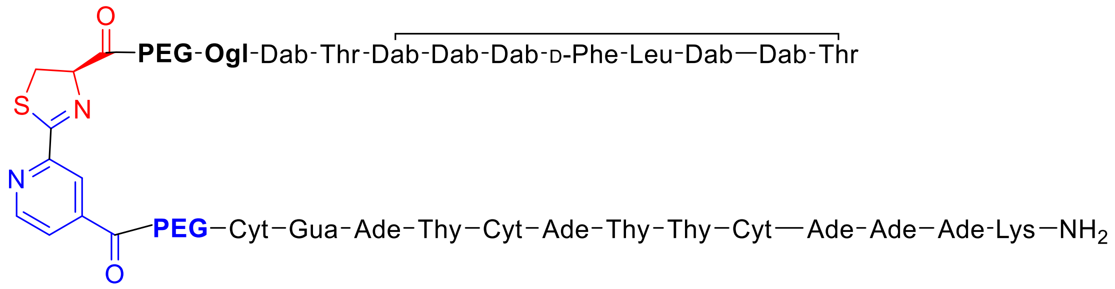

LASP-130 was purified by method B and analyzed by method C. Formic acid salt was obtained in a yield of 16 mg, retention time (t_R) at 214 nm = 17.660 min (purity: 93.37 %). ESI-MS analysis of peak at 17.66 min: *m/z*, $[M+3H]^{3+}$ 1703.00, $[M+4H]^{4+}$ 1277.5, $[M+5H]^{5+}$ 1022.2. Calculated mass (monoisotopic) LASP-130 ($C_{214}H_{307}N_{93}O_{56}S_8$) 5104.42.

LASP-131

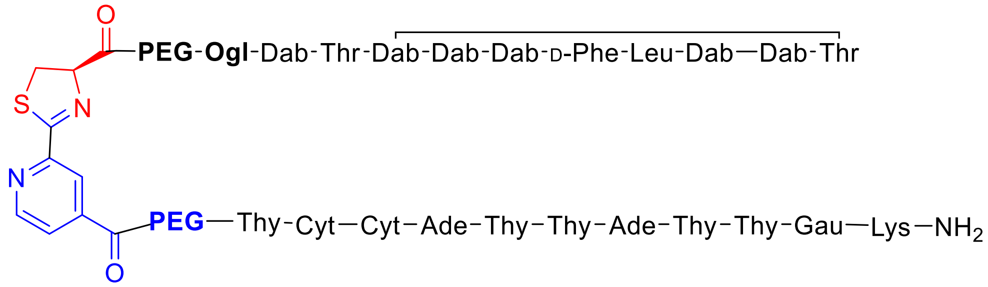

LASP-131 was purified by method B and analyzed by method C. Formic acid salt was obtained in a yield of 13 mg, retention time (t_R) at 214 nm = 17.520 min (purity: 95.04 %). ESI-MS analysis of peak at 17.52 min: *m/z*, $[M+3H]^{3+}$ 1521.40, $[M+4H]^{4+}$ 1141.25, $[M+5H]^{5+}$ 913.25, $[M+6H]^{6+}$ 761.20. Calculated mass (monoisotopic) LASP-131 ($C_{193}H_{283}N_{75}O_{55}S$) 4560.87.

LASP-132

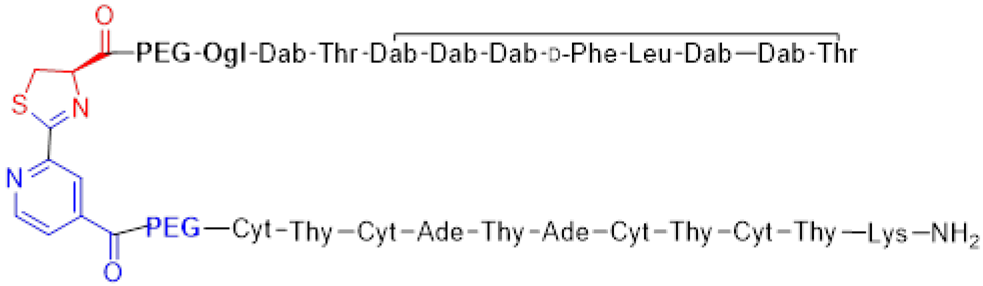

2

LASP-132 was purified by method B and analyzed by method C. Formic acid salt was obtained in a yield of 11 mg, retention time (t_R) at 214 nm = 17.191 min (purity: 92.97 %). ESI-MS analysis of peak at 17.19 min: *m/z*, $[M+3H]^{3+}$ 1503.05, $[M+4H]^{4+}$ 1127.45, $[M+5H]^{5+}$ 902.25, $[M+6H]^{6+}$ 752.05. Calculated mass (monoisotopic) for LASP-132 ($C_{191}H_{282}N_{74}O_{54}S$) 4506.83.

LASP-133

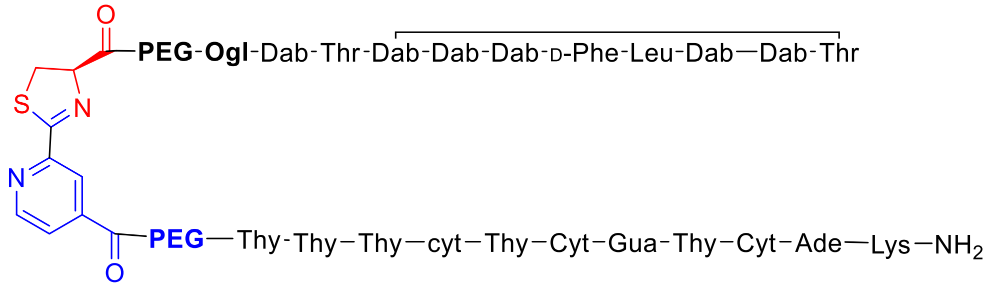

LASP-133 was purified by method B and analyzed by method C. TFA salt was obtained in a yield of 8.3 mg, retention time (t_R) at 214 nm = 17.651 in (purity: 95.96 %). ESI-MS analysis of peak at 17.65 min: *m/z*, $[M+3H]^{3+}$ 1513.35, $[M+4H]^{4+}$ 1135.20 $[M+5H]^{5+}$ 908.40, $[M+6H]^{6+}$ 757.15. Calculated mass (monoisotopic) for LASP-133 ($C_{192}H_{282}N_{73}O_{56}S$) 4536.83.

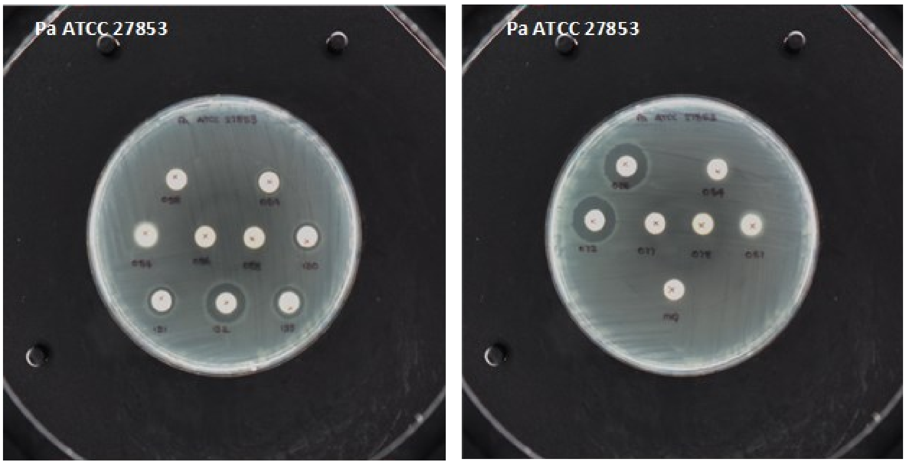

Figure S7: The inhibition zone (mm) of all peptides, PNA and peptide-PNA conjugates against *P. aeruginosa* ATCC 27853 at a concentration of 40 µM.

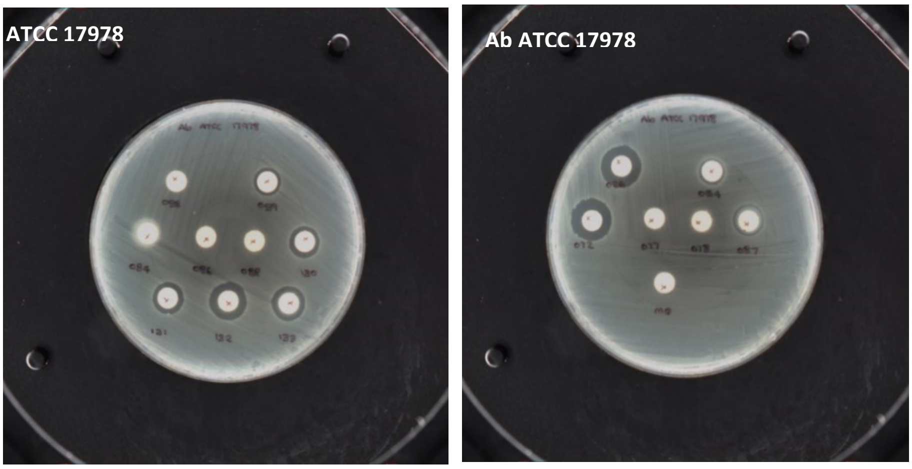

Figure S8: The inhibition zone (mm) of all peptides, PNA and peptide-PNA conjugates against *A. baumannii* ATCC 17978 at a concentration of 40 µM.

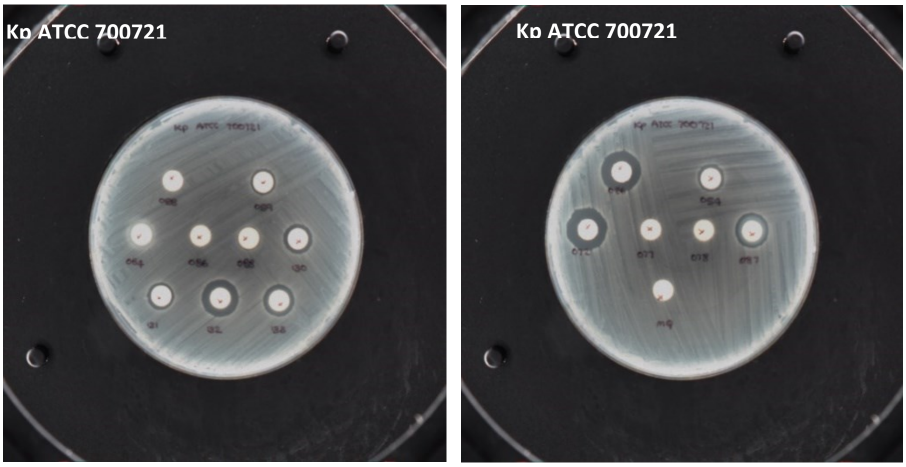

Figure S9: The inhibition zone (mm) of all peptides, PNA and peptide-PNA conjugates against *Klebsiella pneumoniae* ATCC 700721 at a concentration of 40 µM.

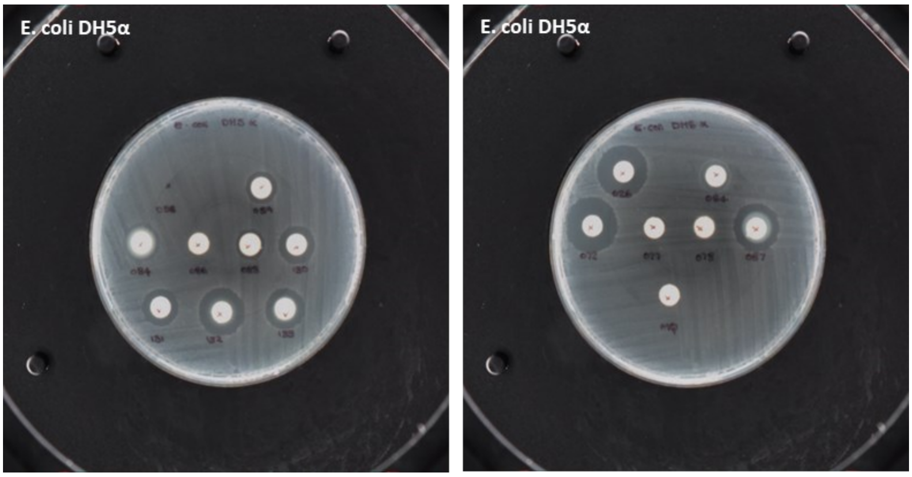

Figure S10: The inhibition zone (mm) of all peptides, PNA and peptide-PNA conjugates against *E. coli* DH5α at a concentration of 40 µM.

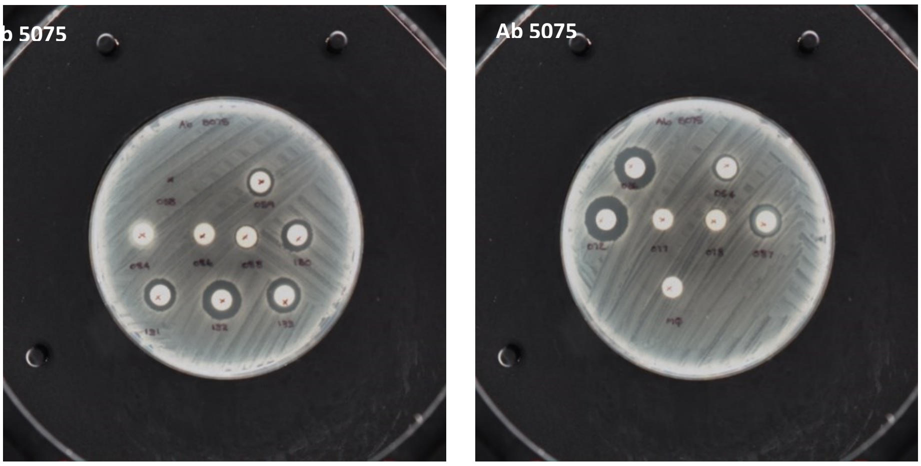

Figure S11: The inhibition zone (mm) of all peptides, PNA and peptide-PNA conjugates against *A. baumannii* 5075 at a concentration of 40 µM.

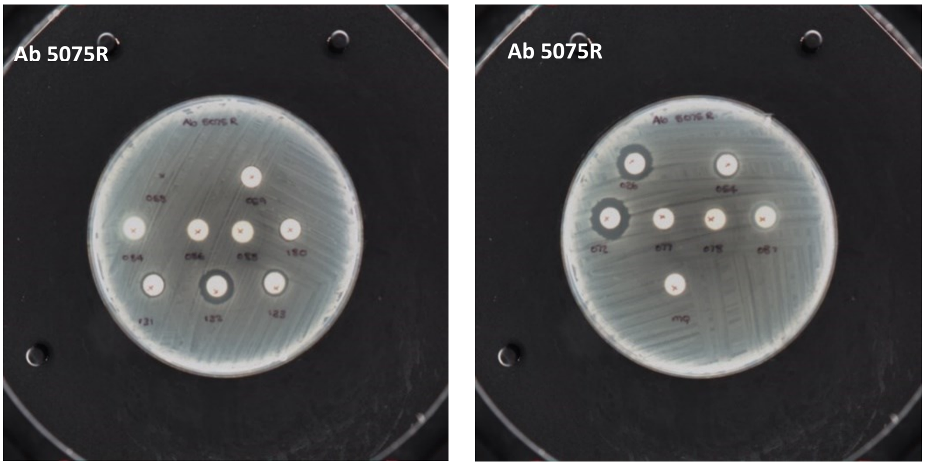

Figure S12: The inhibition zone (mm) of all peptides, PNA and peptide-PNA conjugates against *A. baumannii* 5075R at a concentration of 40 µM.

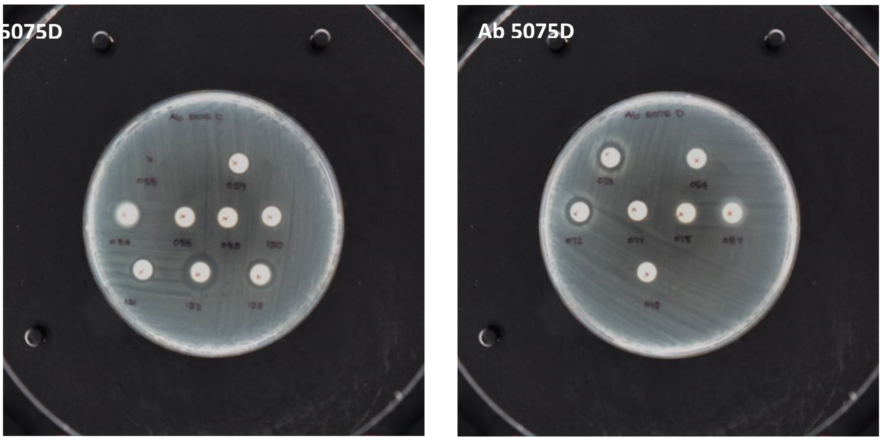

Figure S13: The inhibition zone (mm) of all peptides, PNA and peptide-PNA conjugates against *A. baumannii* 5075D at a concentration of 40 µM.

HPLC and MS Data

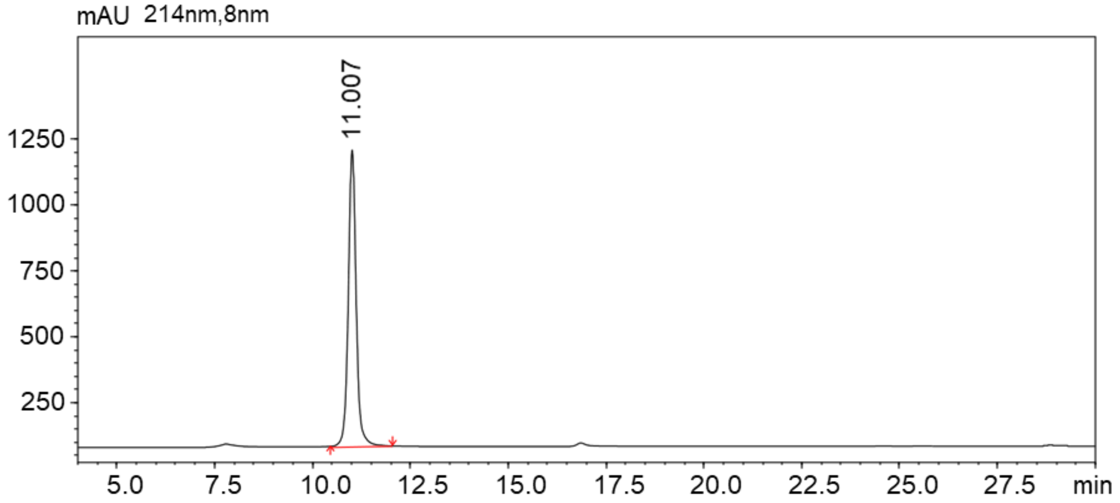

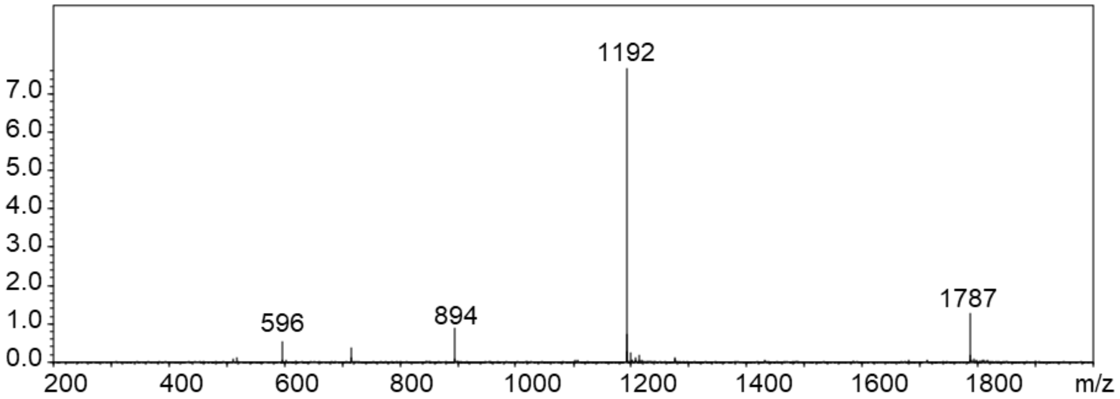

Figure S14. LC-MS Profile for LASP-050 (Method D). (A) LC profile at 214 nm. (B) MS spectrum of the peak at 11.007 min.

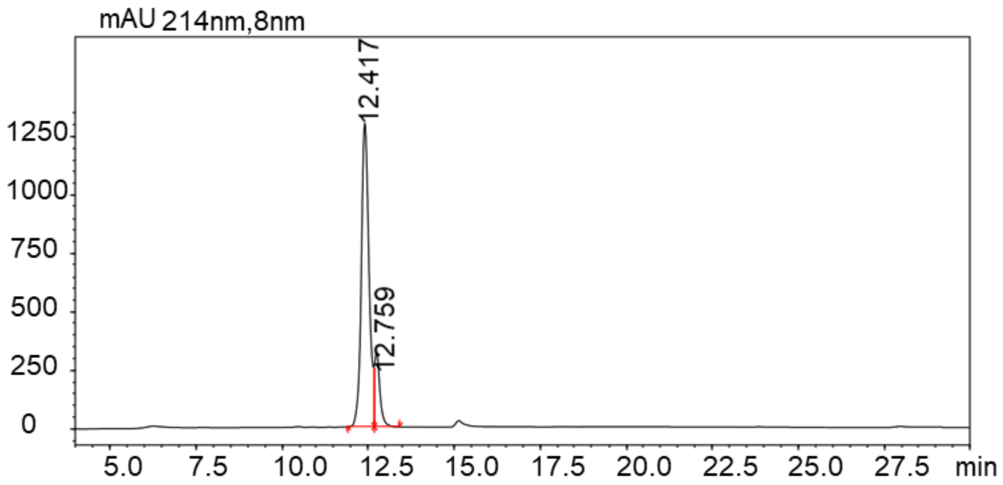

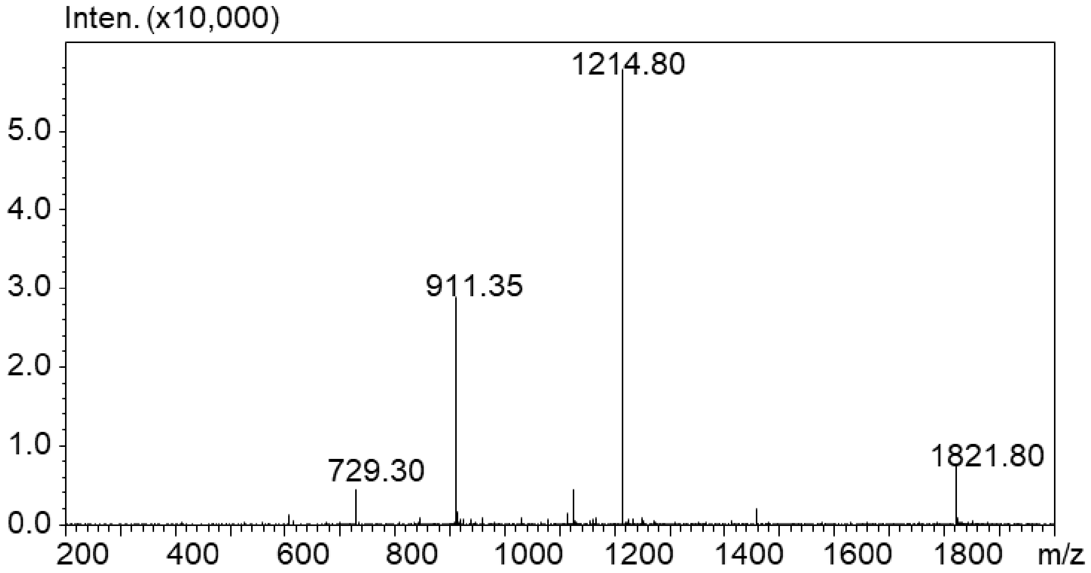

Figure S15. LC-MS Profile for LASP-095 (Method D). (A) LC profile at 214 nm. (B) MS spectrum of the peak at 12.417 min.

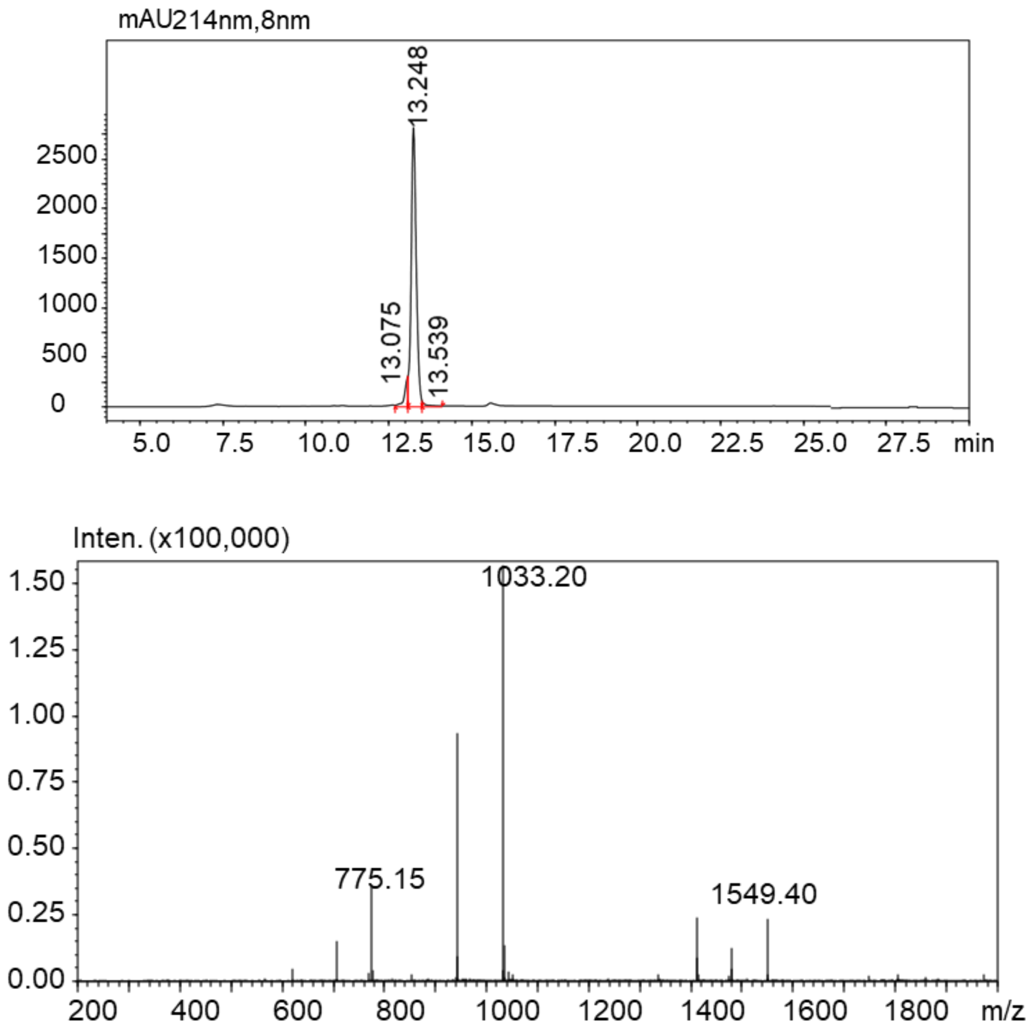

Figure S16. LC-MS Profile for LASP-096 (Method D). (A) LC profile at 214 nm. (B) MS spectrum of the peak at 13.248 min.

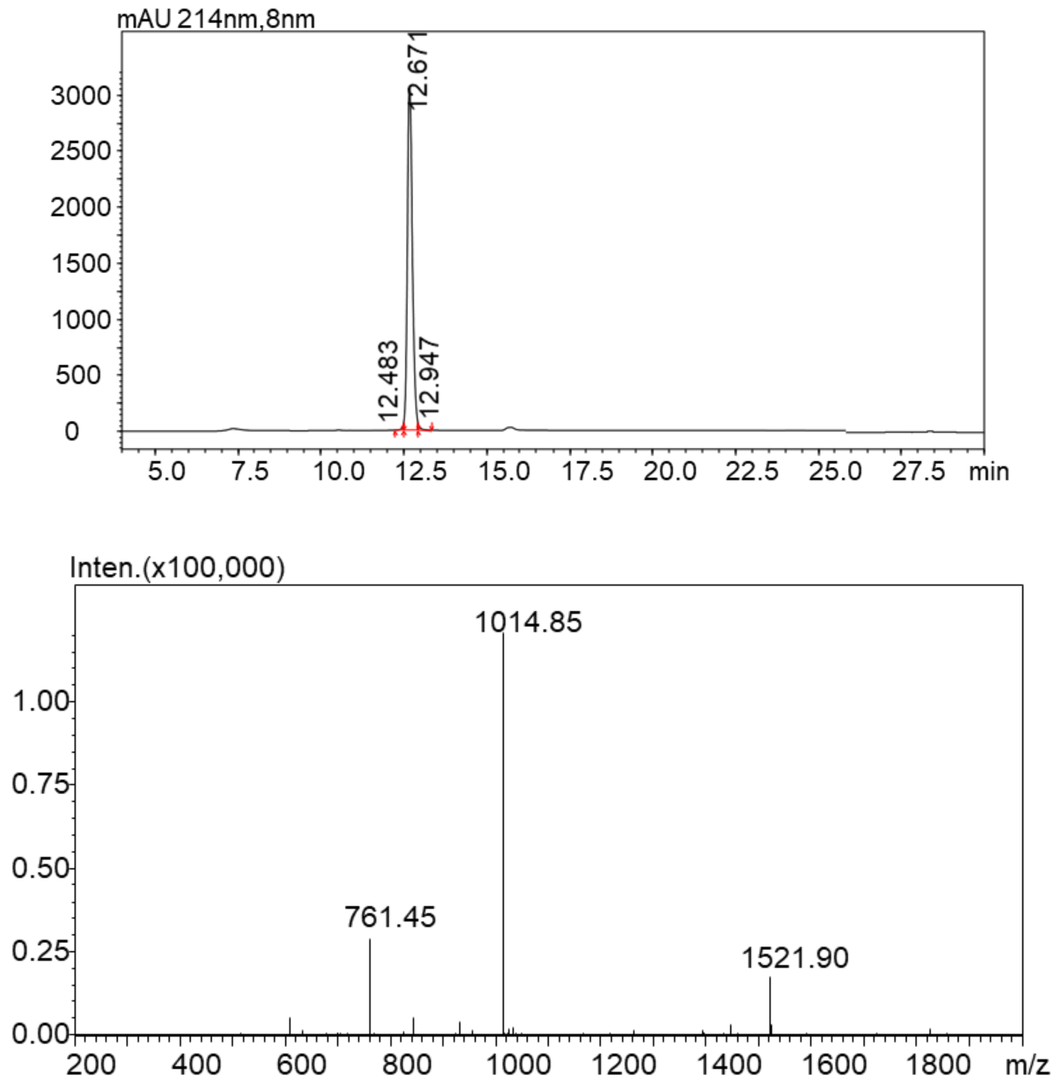

Figure S17. LC-MS Profile for LASP-097 (Method D). (A) LC profile at 214 nm. (B) MS spectrum of the peak at 12.671 min.

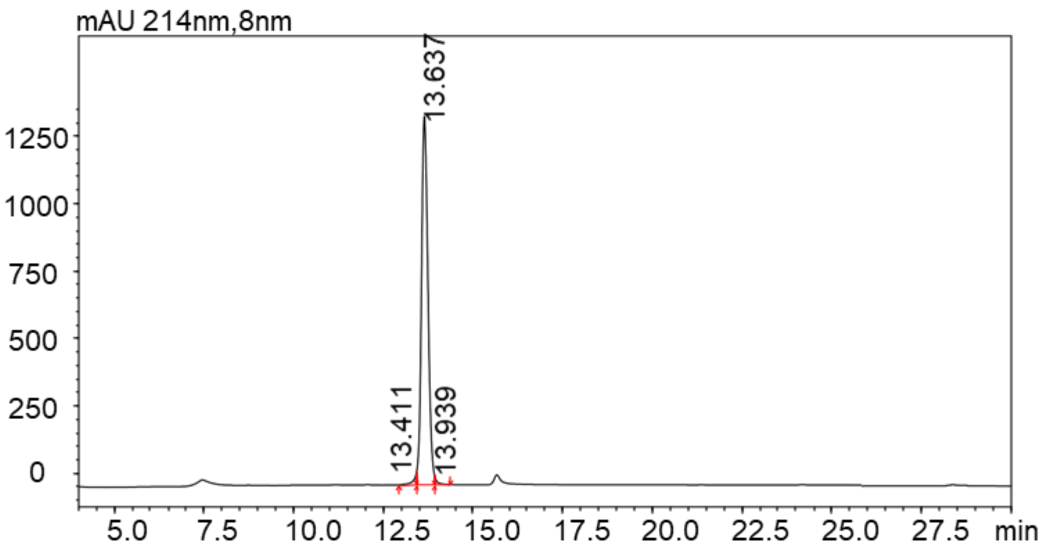

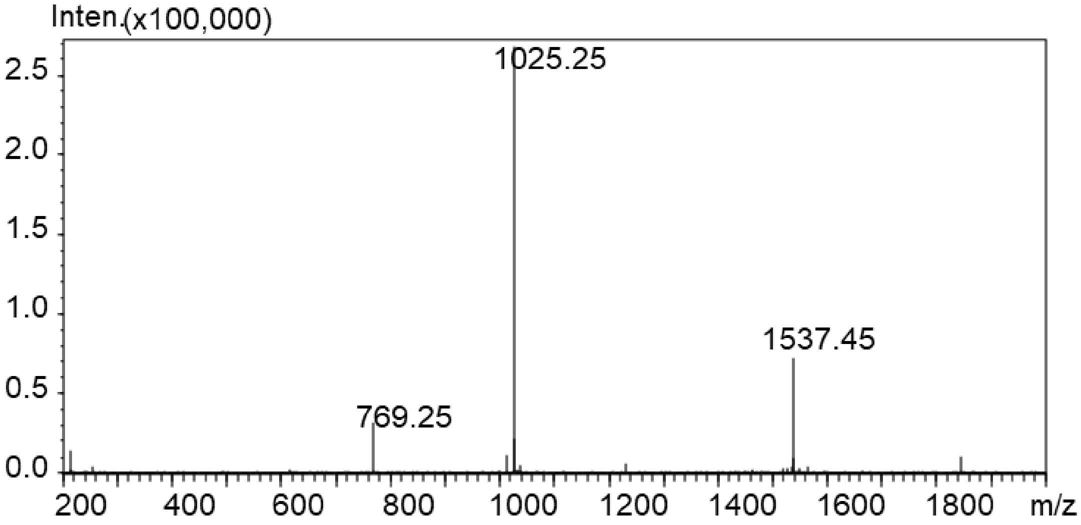

Figure S18. LC-MS Profile for LASP-119 (Method D). (A) LC profile at 214 nm. (B) MS spectrum of the peak at 13.637 min.

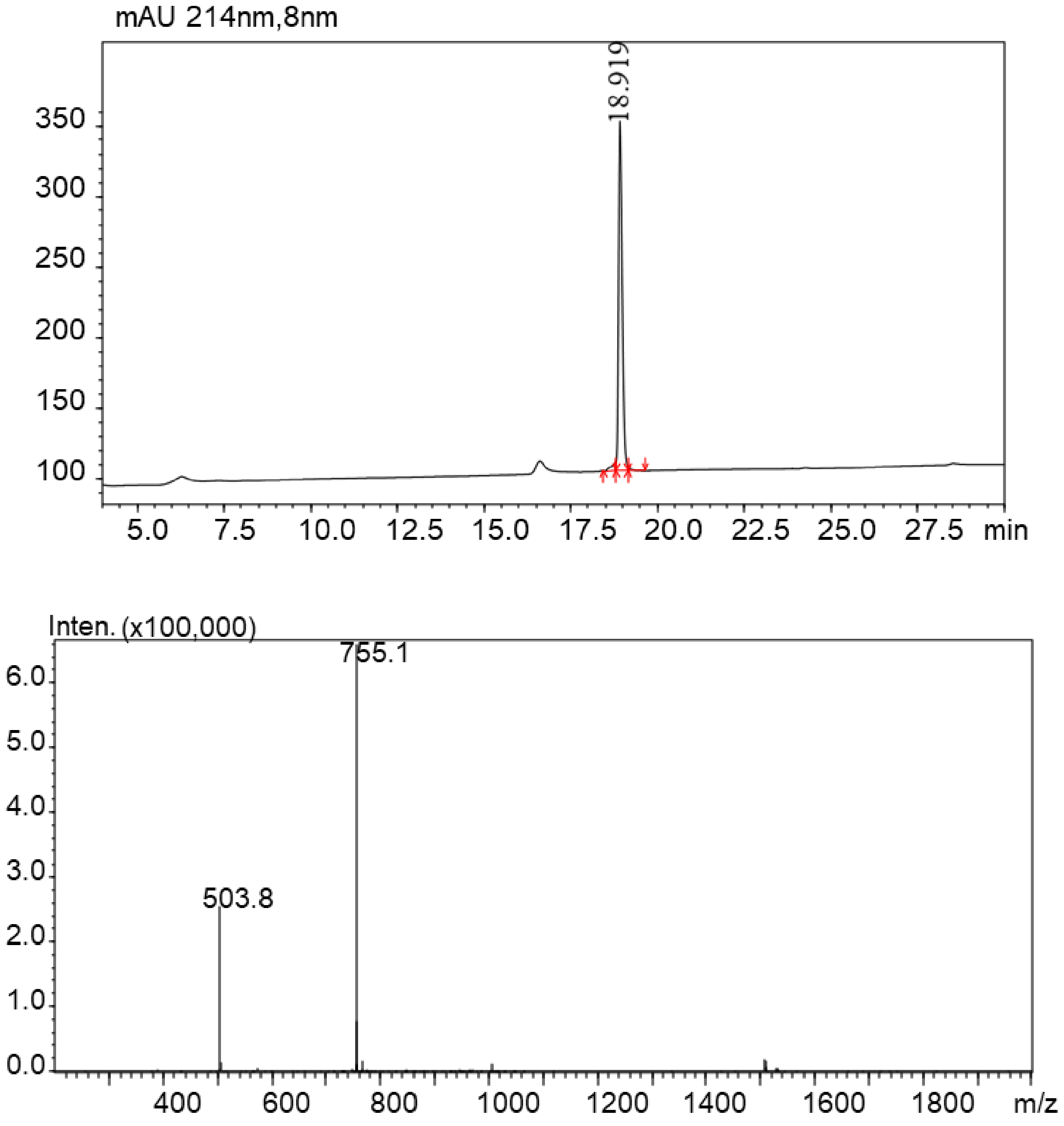

Figure S19. LC-MS Profile for LASP-026 (Method D). (A) LC profile at 214 nm. (B) MS spectrum of the peak at 13.621 min.

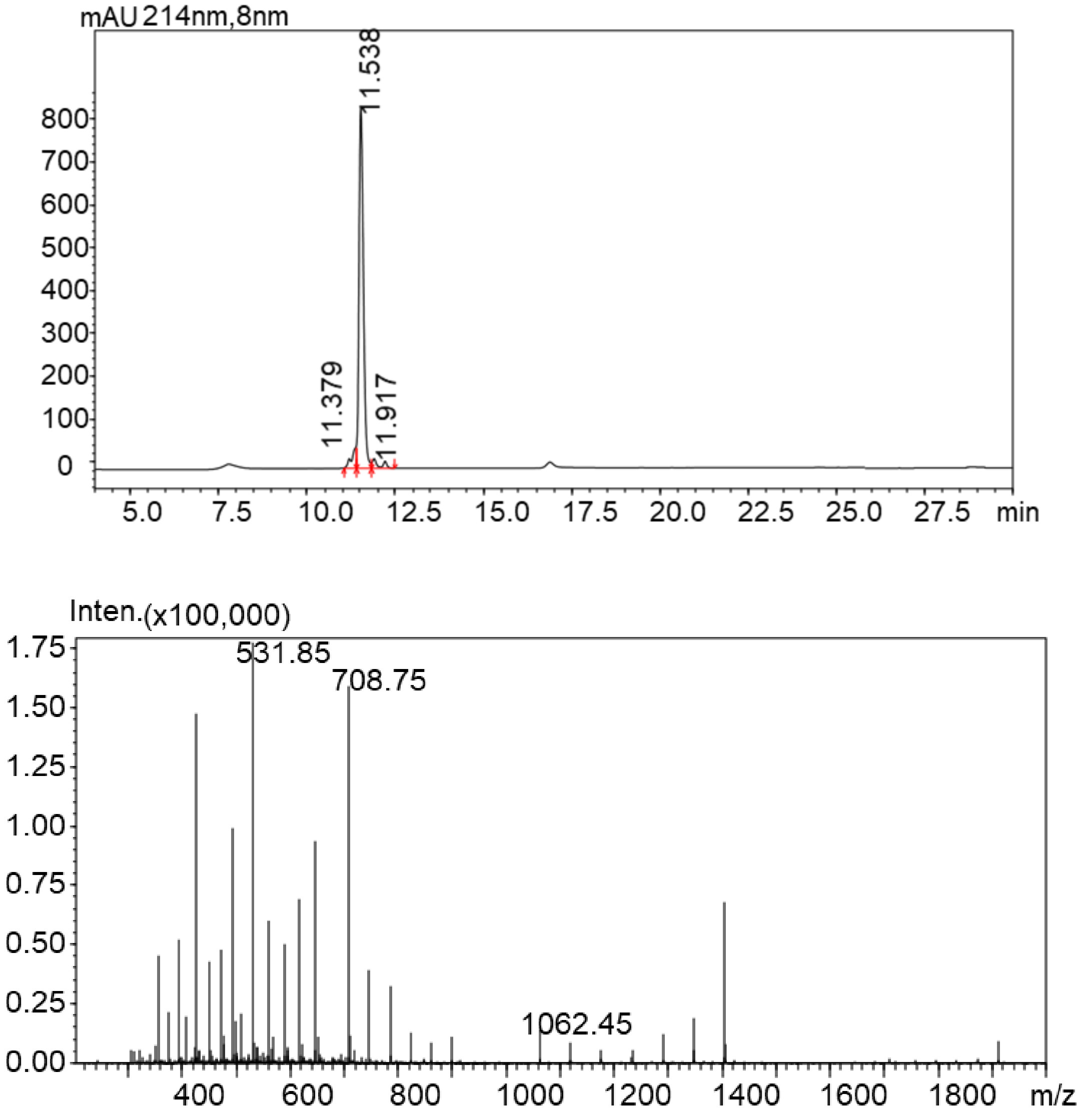

Figure S20. LC-MS Profile for LASP-054 (Method D). (A) LC profile at 214 nm. (B) MS spectrum of the peak at 11.538 min.

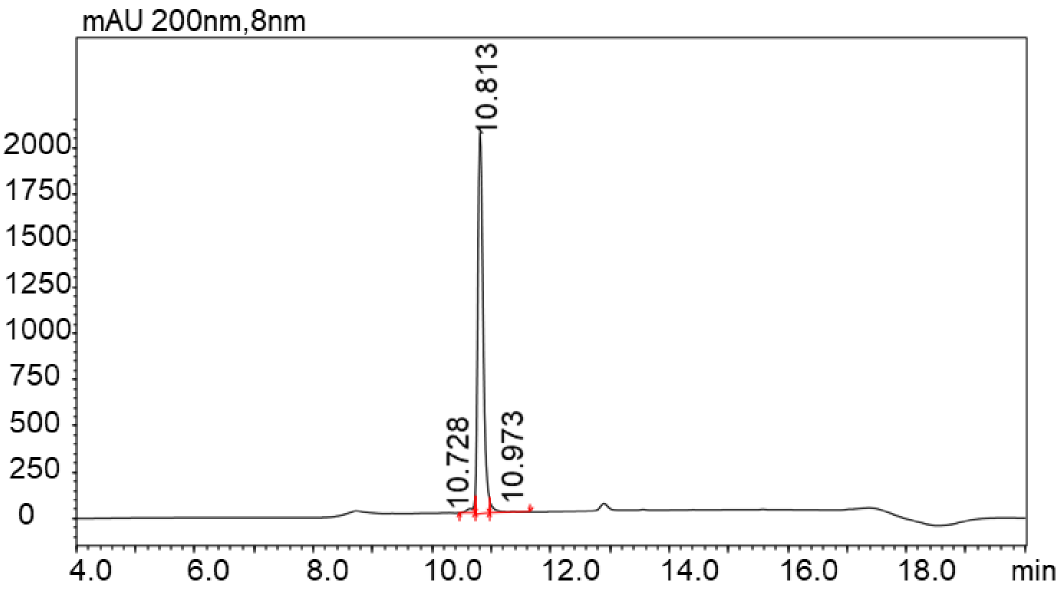

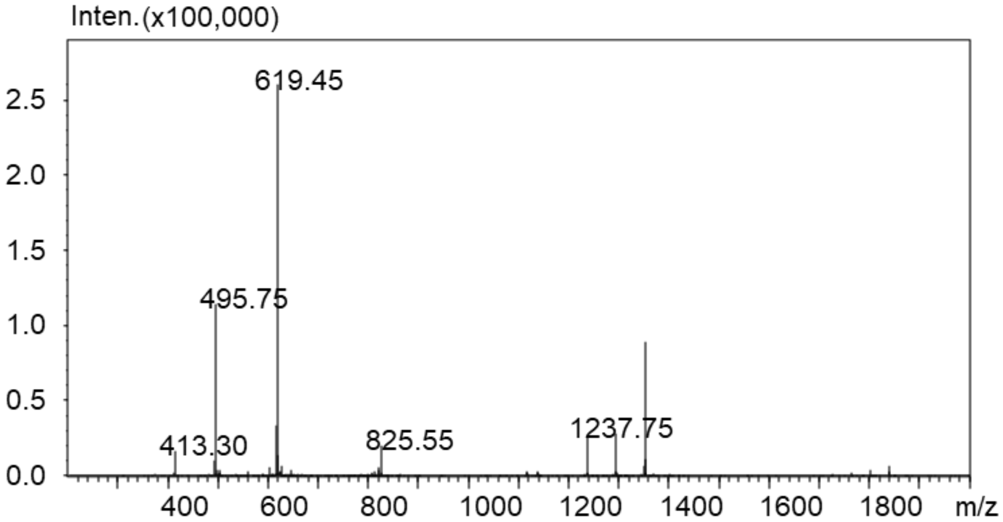

Figure S21. LC-MS Profile for LASP-077 (Method C). (A) LC profile at 214 nm. (B) MS spectrum of the peak at 10.813 min.

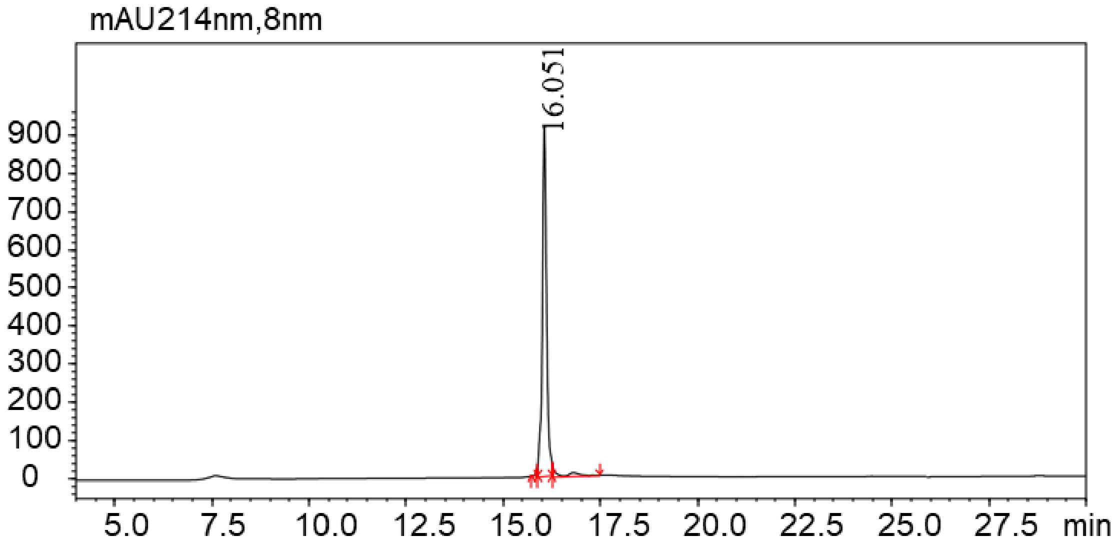

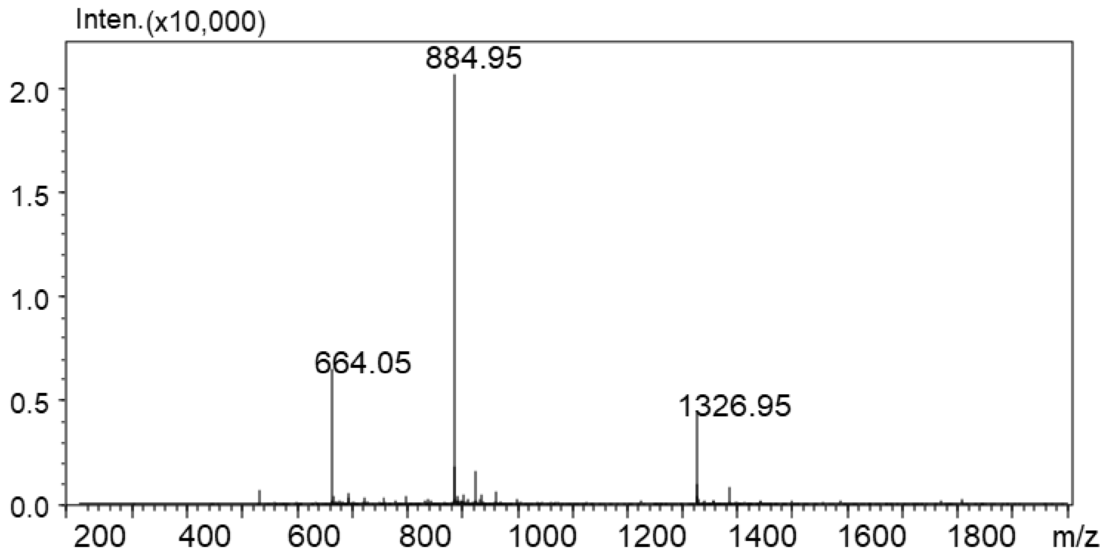

Figure S22. LC-MS Profile for LASP-078 (Method D). (A) LC profile at 214 nm. (B) MS spectrum of the peak at 16.051 min.

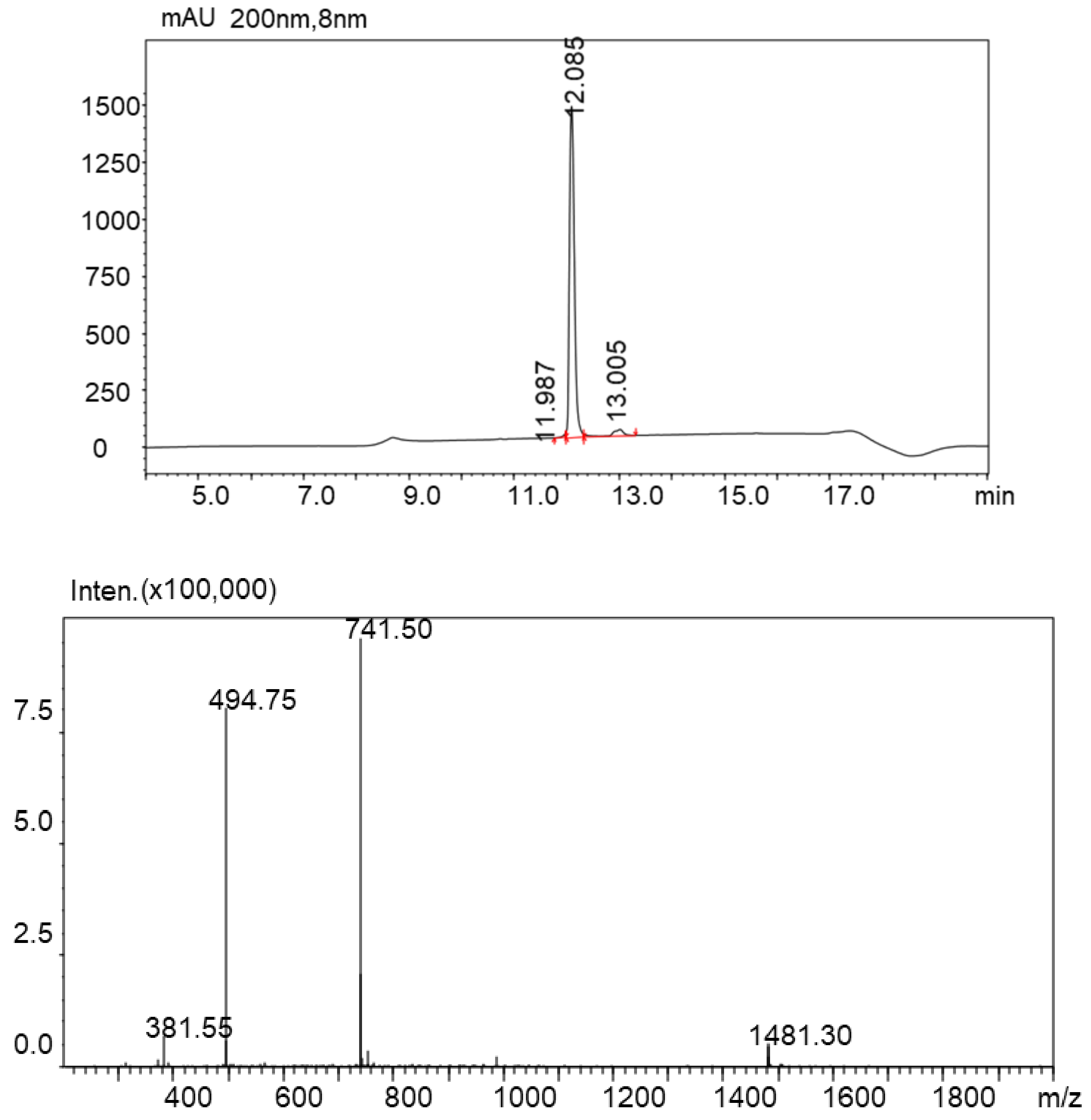

Figure S23. LC-MS Profile for LASP-072 (Method C). (A) LC profile at 214 nm. (B) MS spectrum of the peak at 12.085 min.

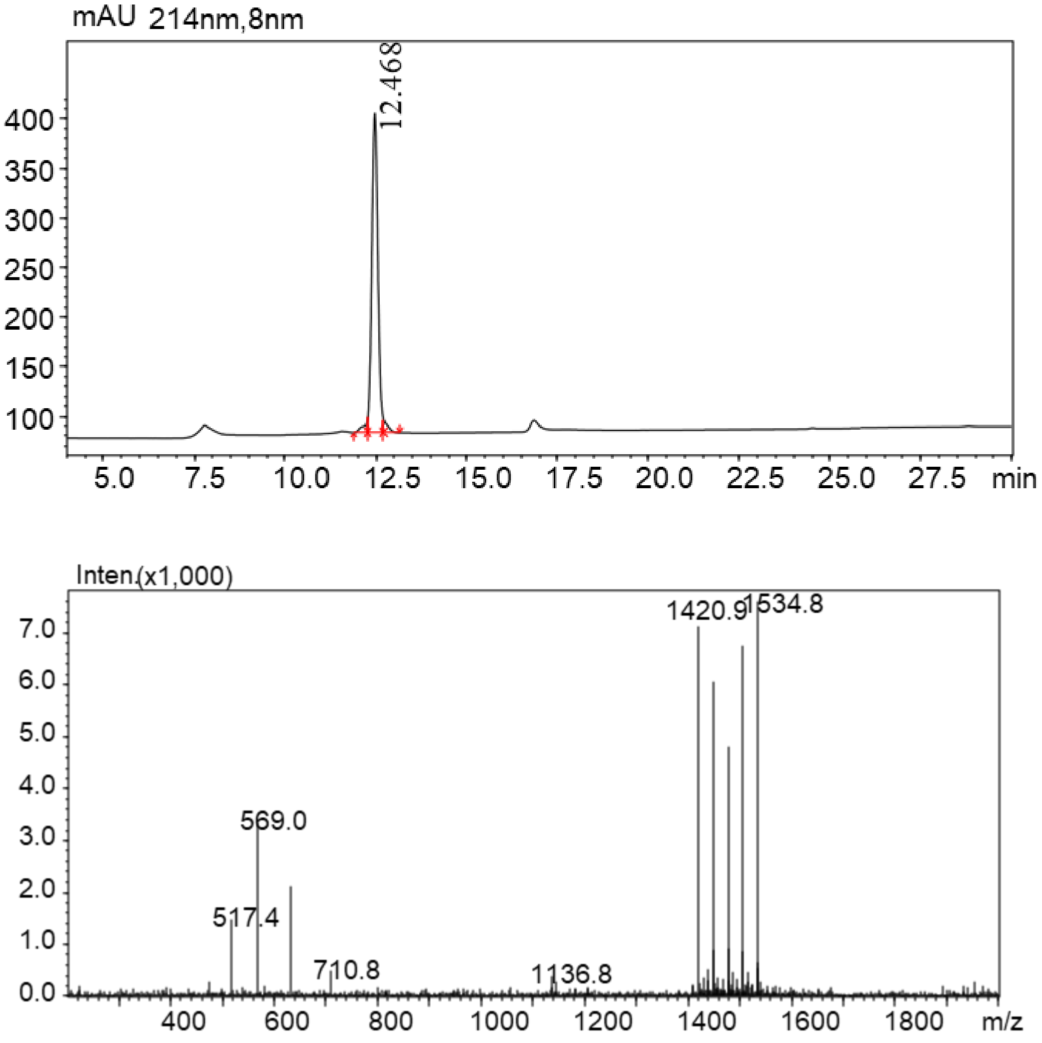

Figure S24. LC-MS Profile for LASP-058 (Method D). (A) LC profile at 214 nm. (B) MS spectrum of the peak at 12.468 min.

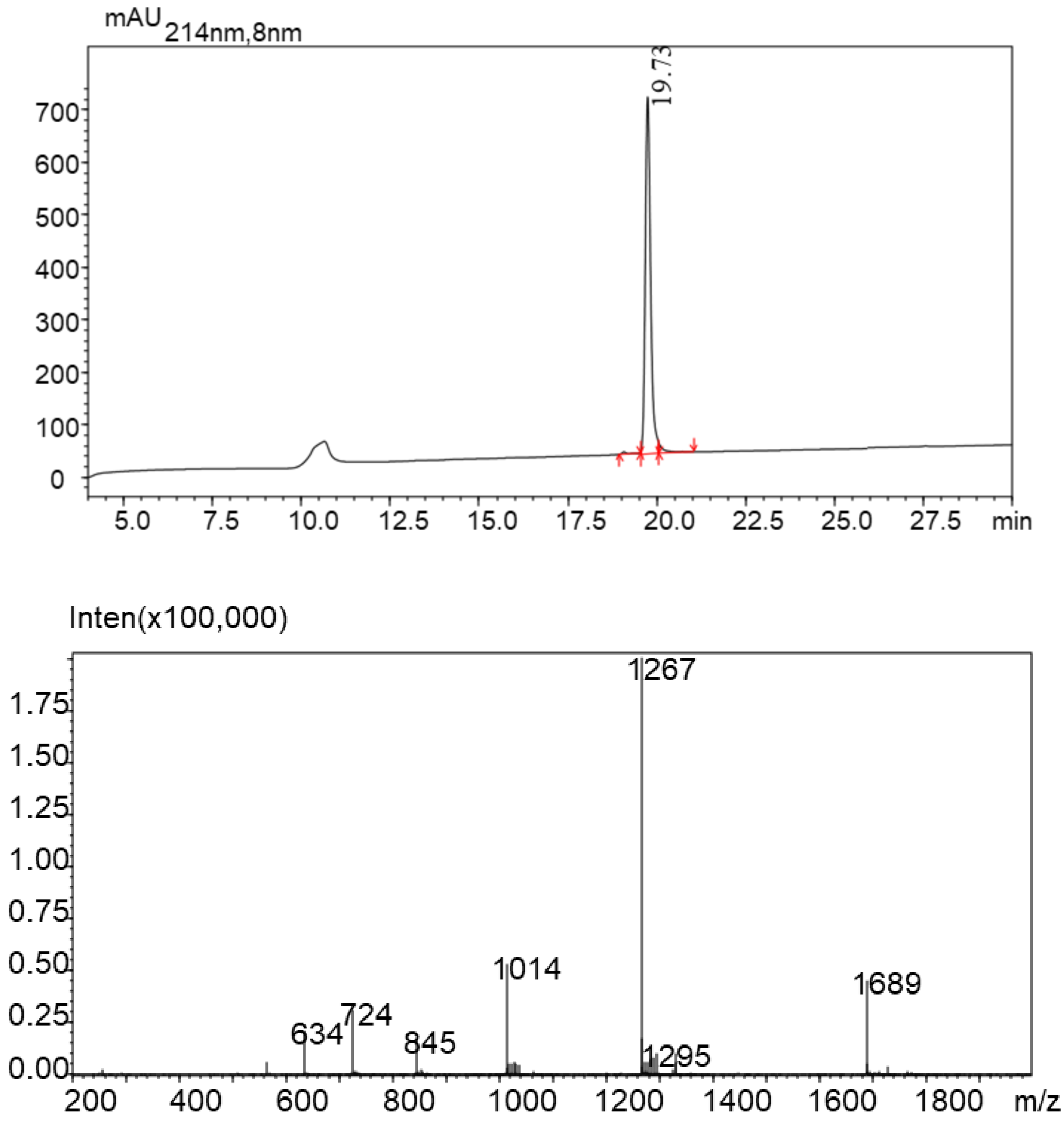

Figure S25. LC-MS Profile for LASP-059 (Method D). (A) LC profile at 214 nm. (B) MS spectrum of the peak at 19.73 min.

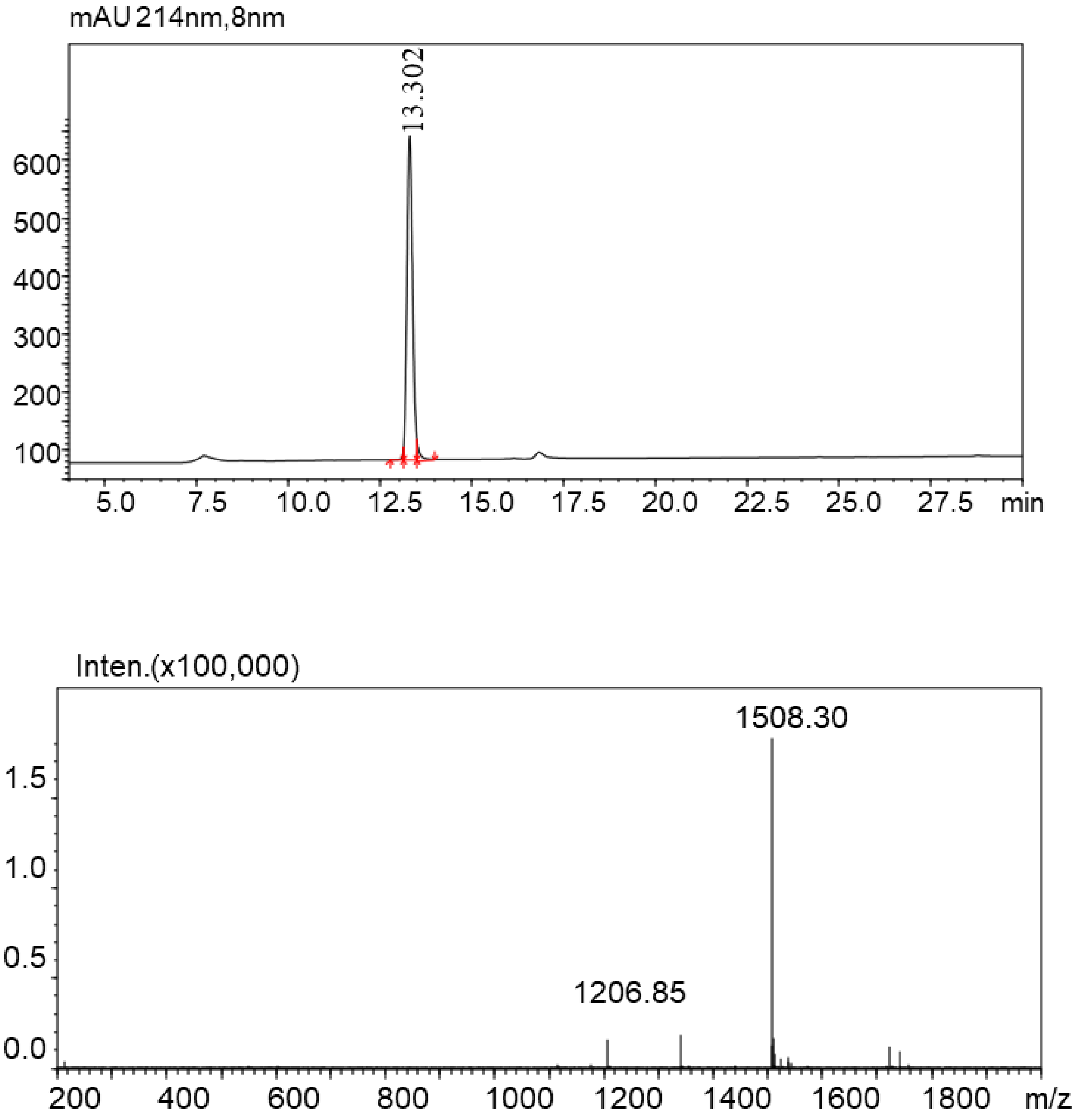

Figure S26. LC-MS Profile for LASP-084 (Method D). (A) LC profile at 214 nm. (B) MS spectrum of the peak at 13.302 min.

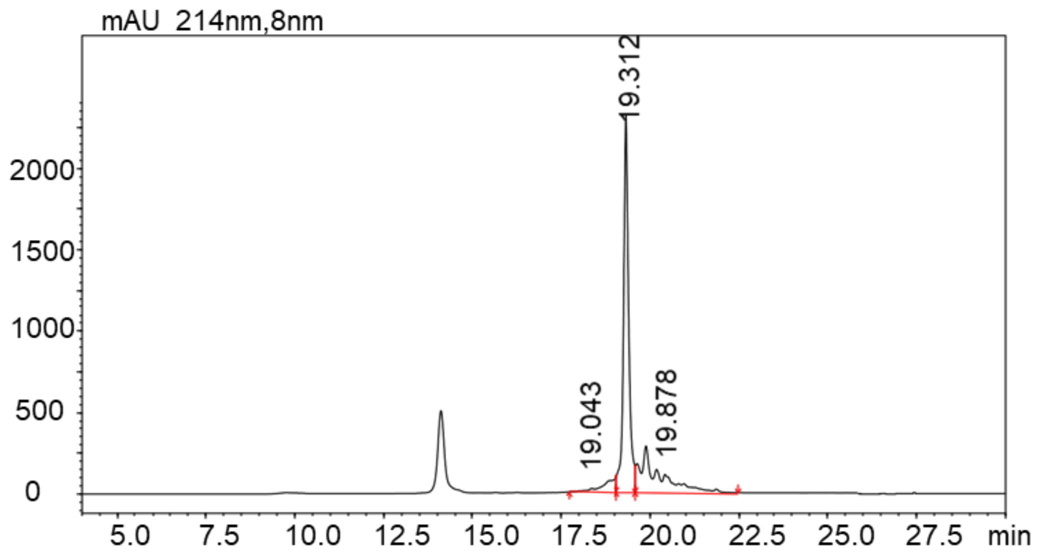

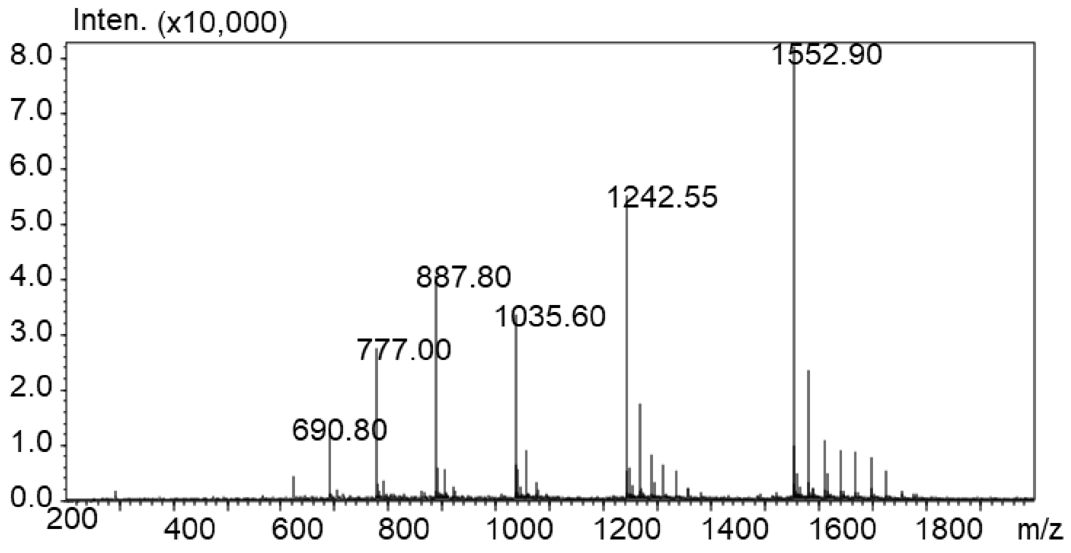

Figure S27. LC-MS Profile for crude LASP-085 (Method D). (A) LC profile at 214 nm. (B) MS spectrum of the peak at 19.312 min.

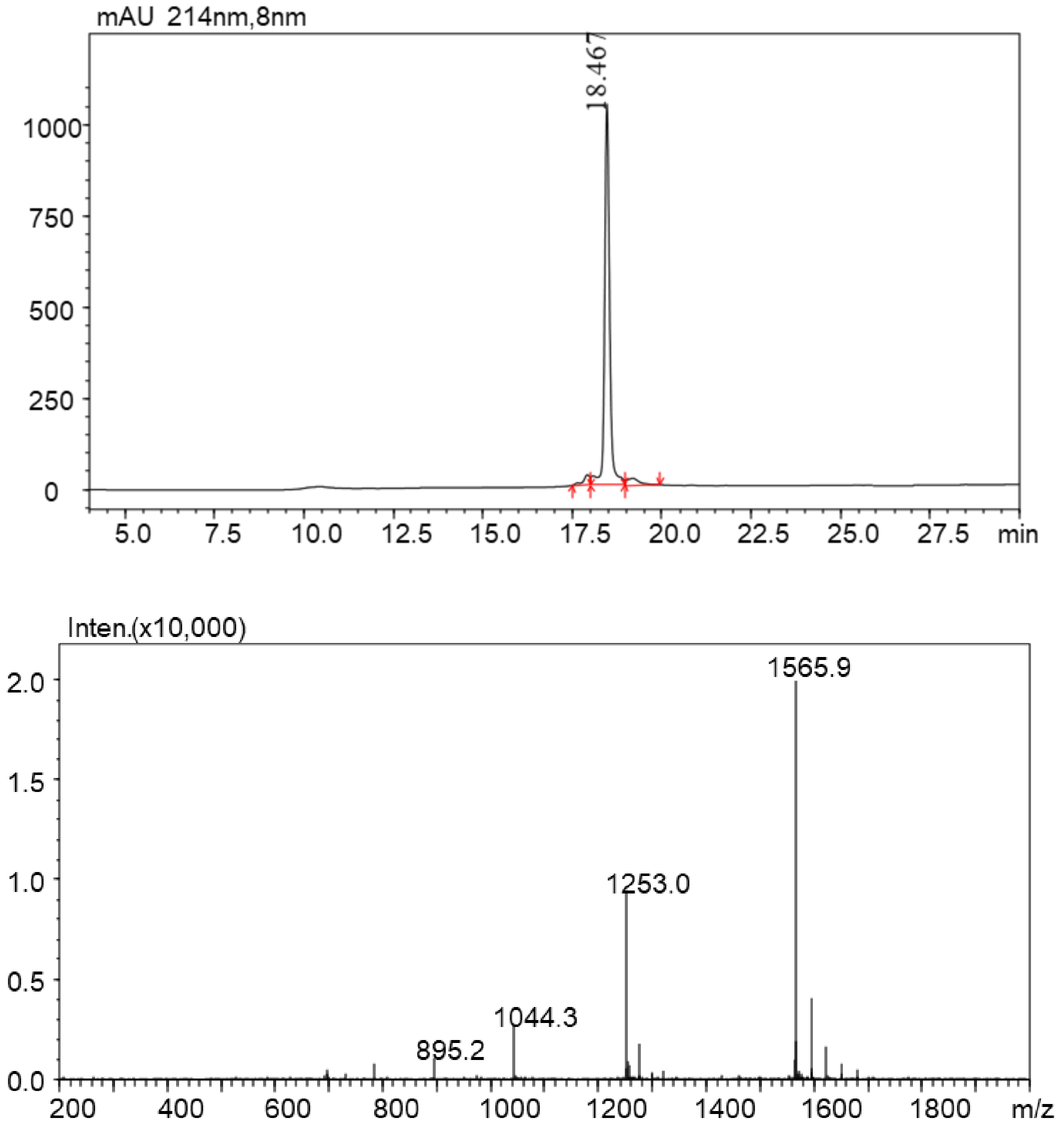

Figure S28. LC-MS Profile for LASP-086 (Method D). (A) LC profile at 214 nm. (B) MS spectrum of the peak at 13.484 min.

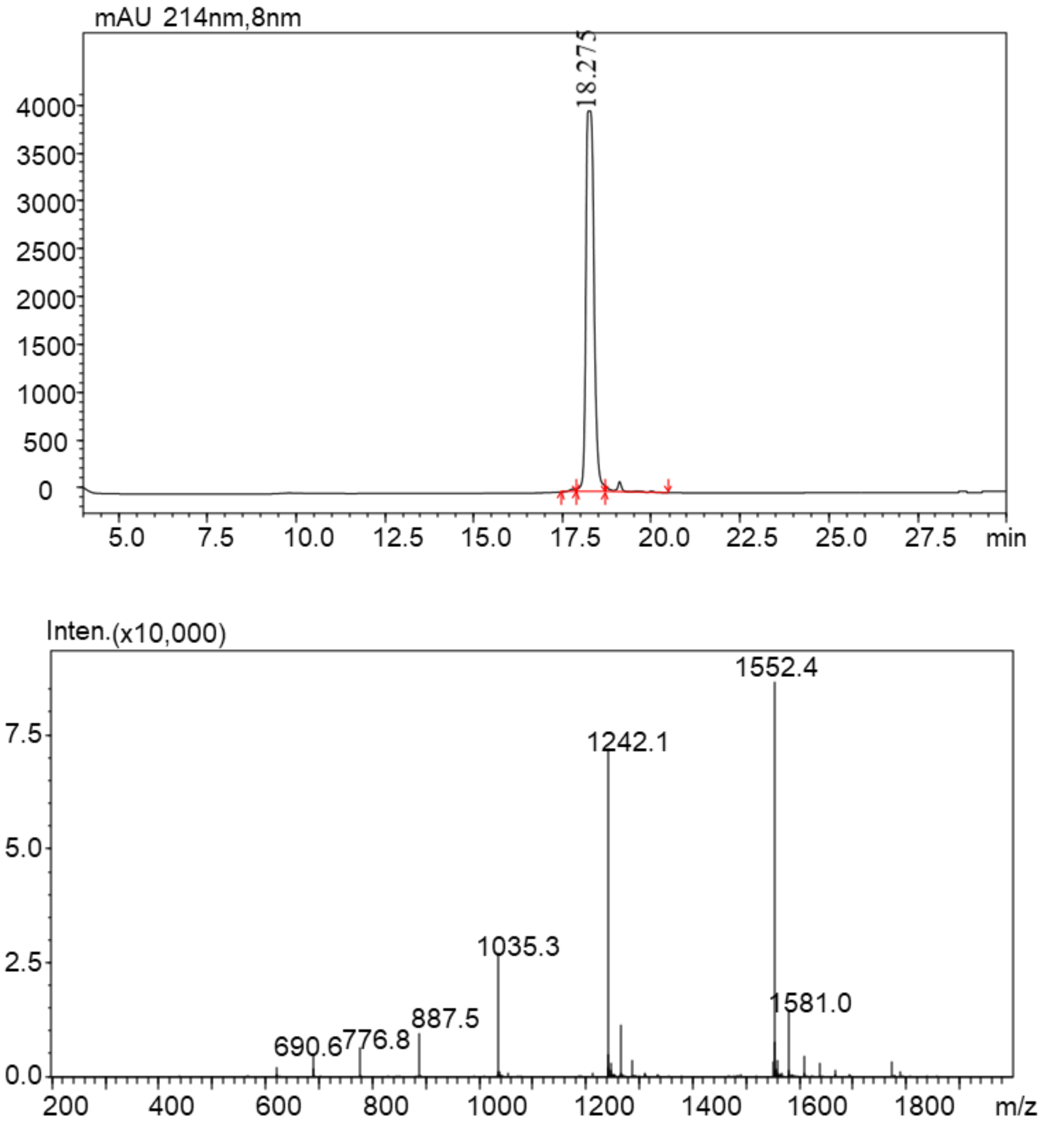

Figure S29. LC-MS Profile for LASP-088 (Method D). (A) LC profile at 214 nm. (B) MS spectrum of the peak at 18.275 min.

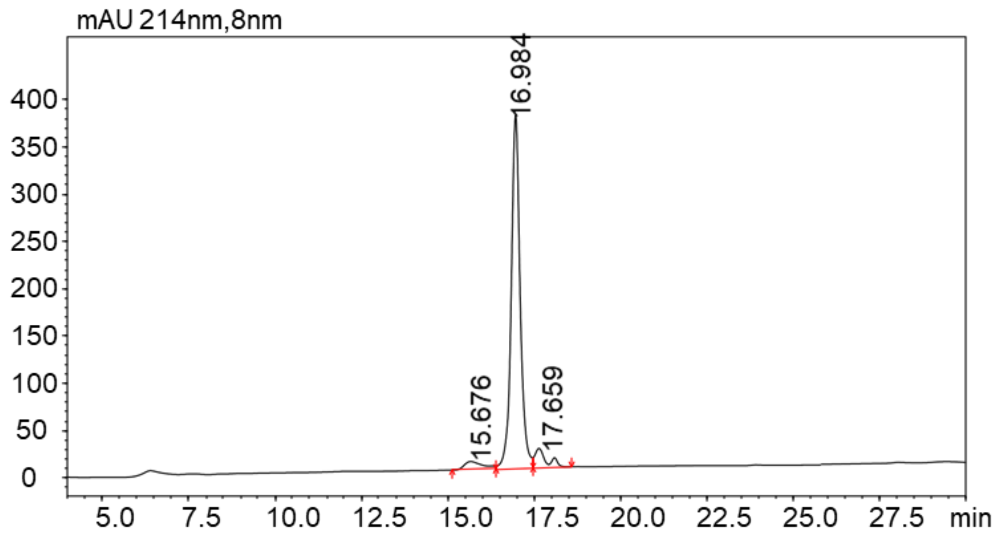

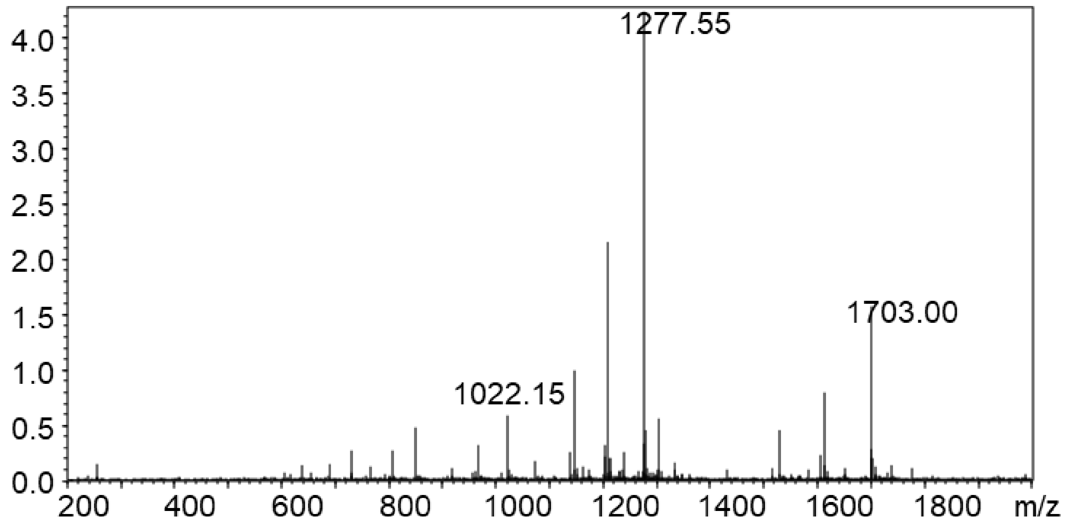

Figure S30. LC-MS Profile for LASP-130 (Method D). (A) LC profile at 214 nm. (B) MS spectrum of the peak at 16.984 min.

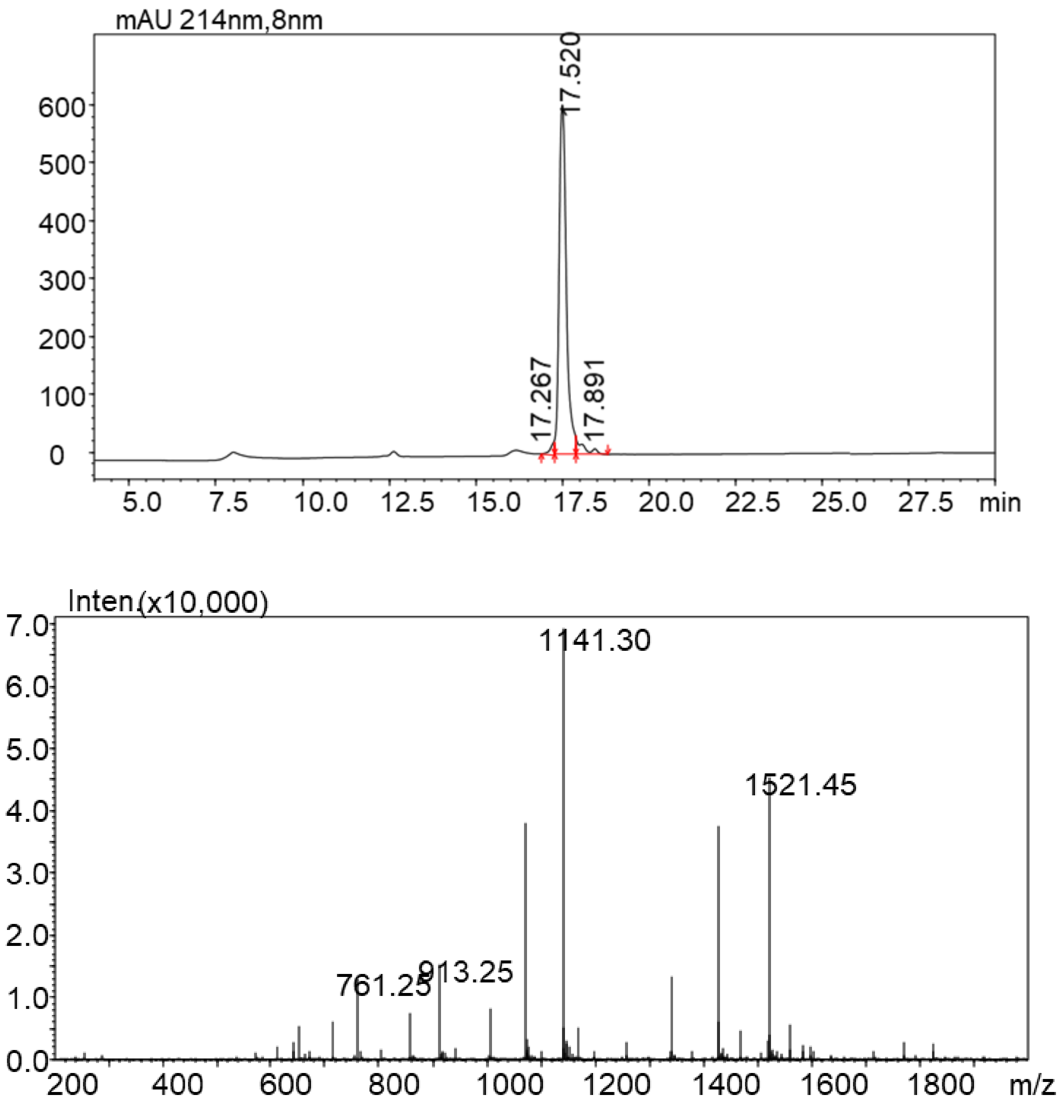

Figure S31. LC-MS Profile for LASP-131 (Method D). (A) LC profile at 214 nm. (B) MS spectrum of the peak at 17.520 min.

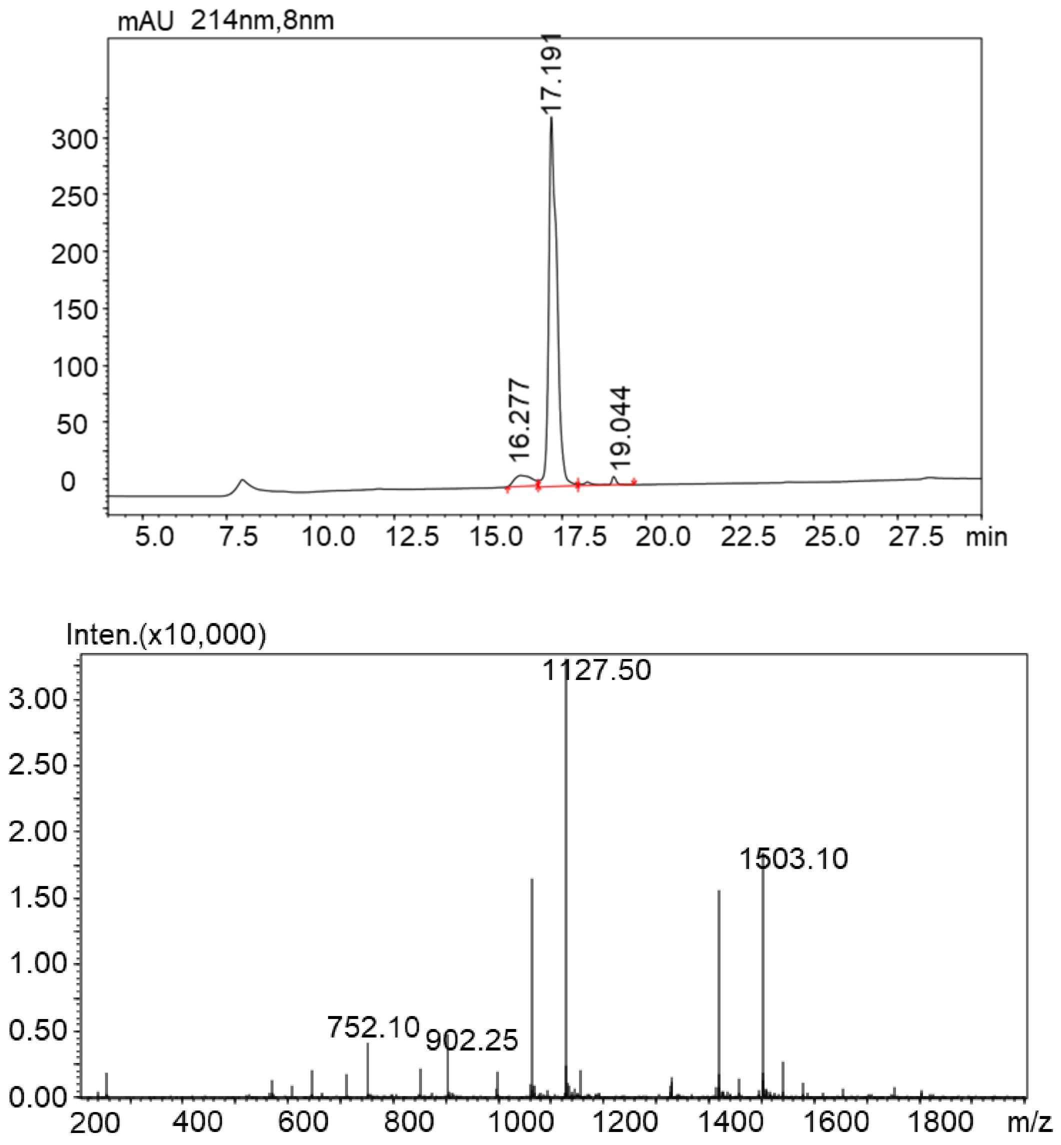

Figure S31. LC-MS Profile for LASP-132 (Method D). (A) LC profile at 214 nm. (B) MS spectrum of the peak at 17.191 min.

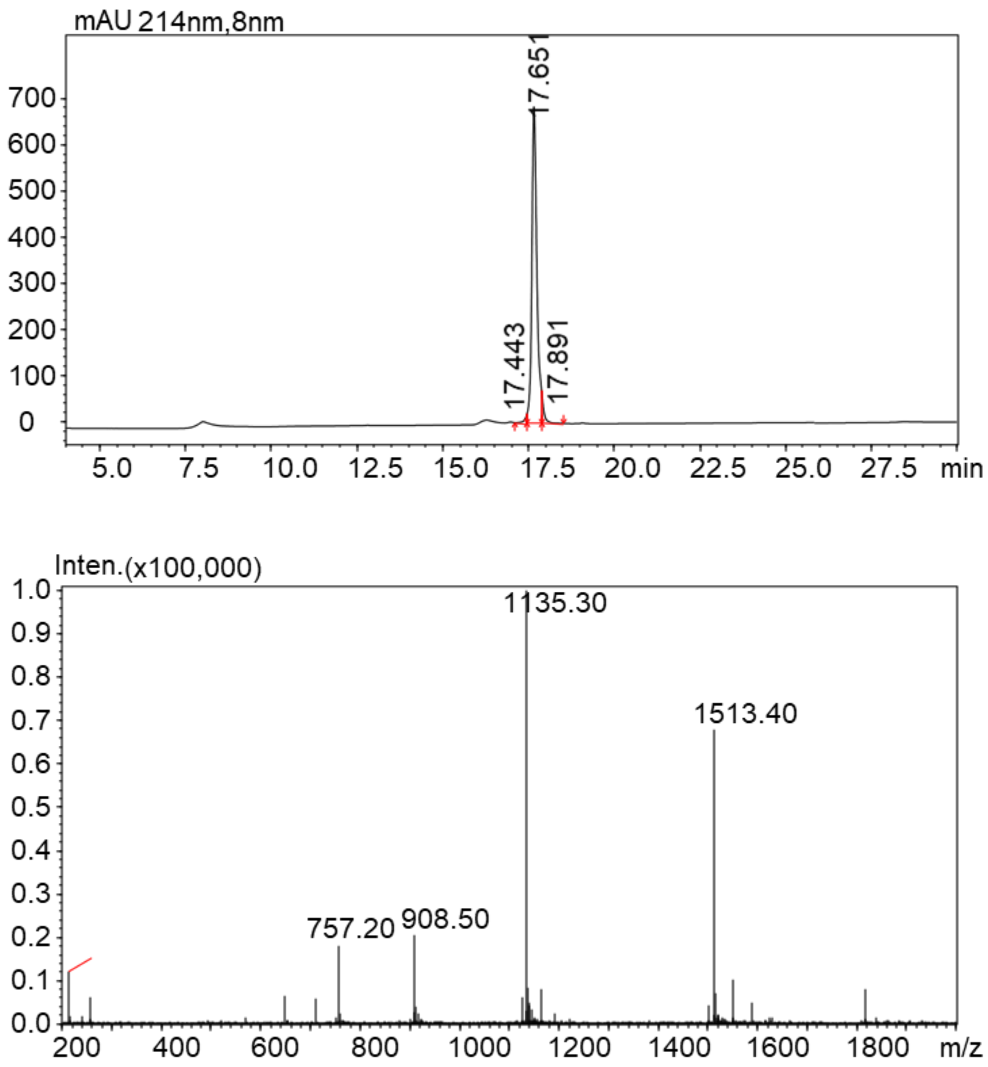

Figure S32. LC-MS Profile for LASP-133 (Method D). (A) LC profile at 214 nm. (B) MS spectrum of the peak at 17.651 min.

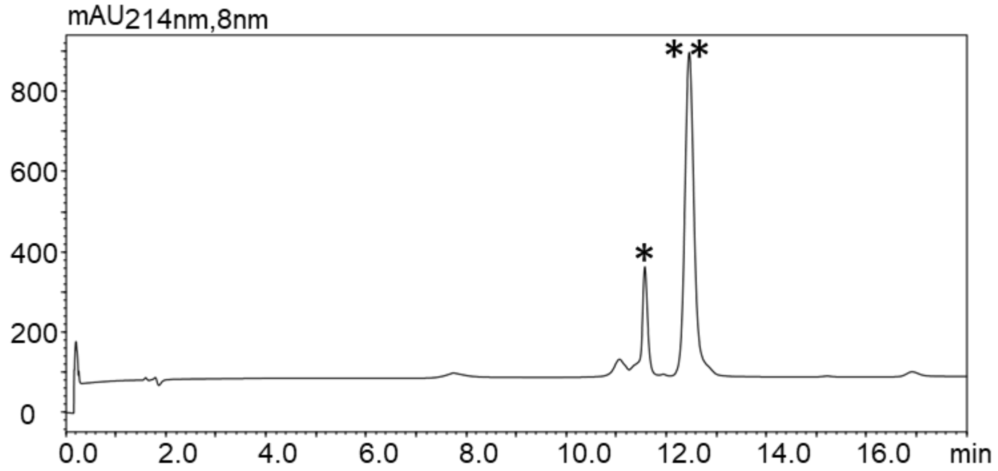

Figure S33. Crude reaction analysis of LASP-058: * Excess peptide, **Conjugated product

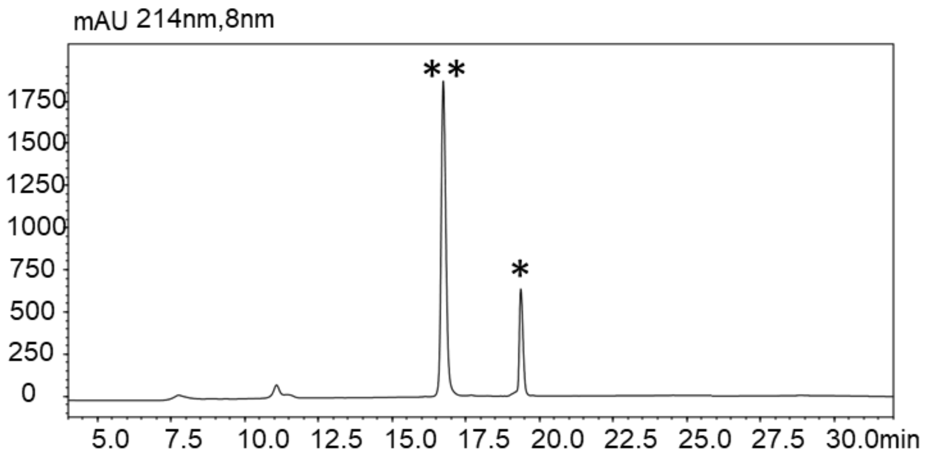

Figure S34. Crude reaction analysis of LASP-059; * Excess peptide, **Conjugated product

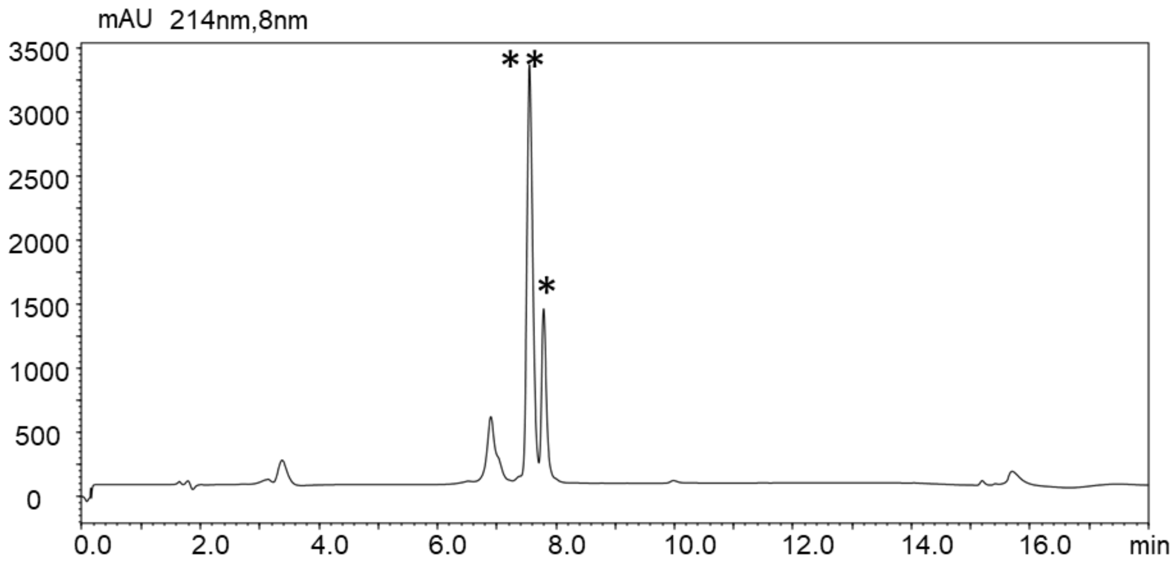

Figure S35. Crude reaction analysis of LASP-084; * Excess peptide, **Conjugated product

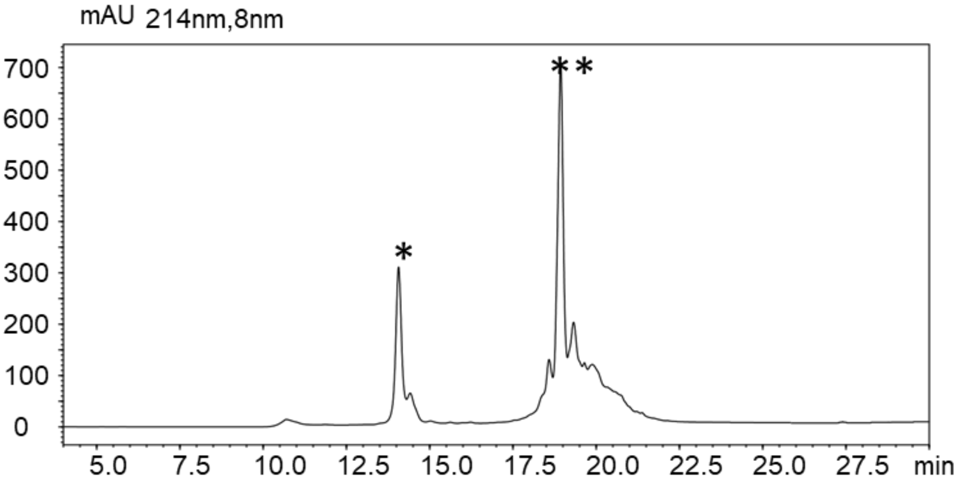

Figure S36. Crude reaction analysis of LASP-086; * Excess peptide, **Conjugated product

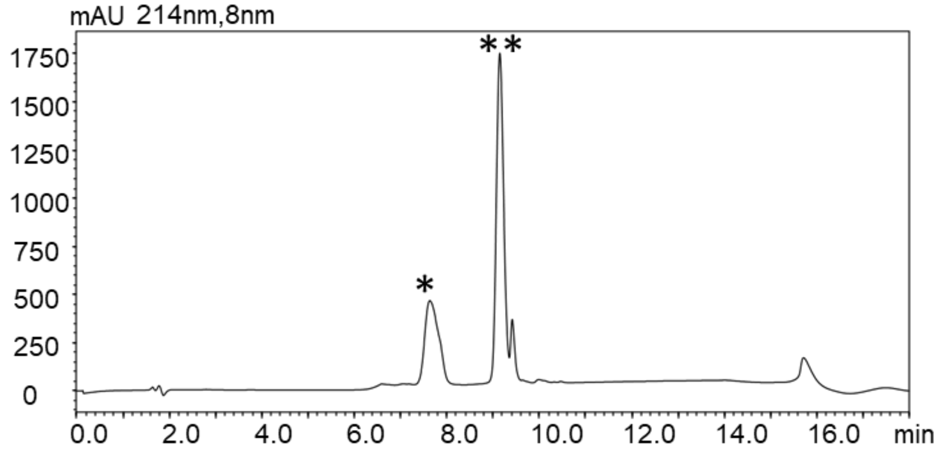

Figure S37. Crude reaction analysis of LASP-130; * Excess peptide, **Conjugated product

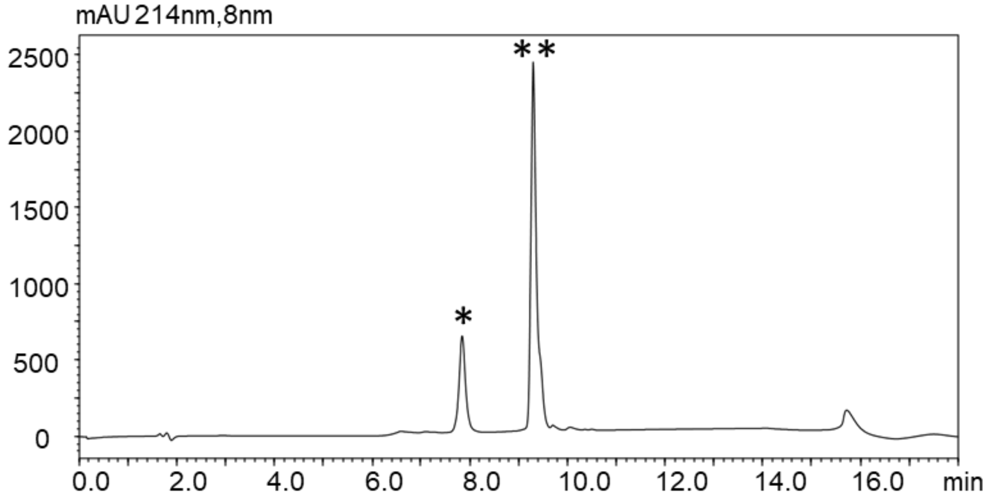

Figure S38. Crude reaction analysis of LASP-131; * Excess peptide, **Conjugated product

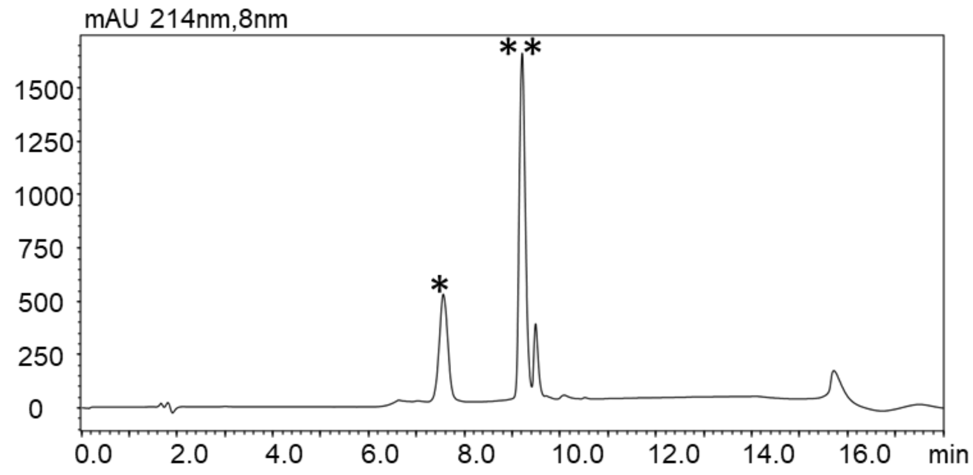

Figure S39. Crude reaction analysis of LASP-132; * Excess peptide, **Conjugated product

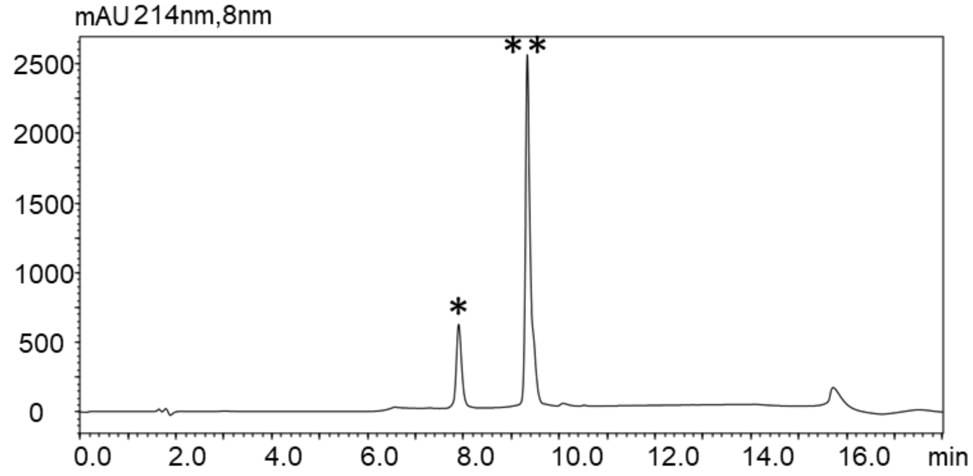

Figure S40 Crude reaction analysis of LASP-133; * Excess peptide, **Conjugated product
